# Three-dimensional tumor growth in time-varying chemical fields: a modeling framework and theoretical study

**DOI:** 10.1186/s12859-019-2997-9

**Published:** 2019-08-27

**Authors:** Markos Antonopoulos, Dimitra Dionysiou, Georgios Stamatakos, Nikolaos Uzunoglu

**Affiliations:** Institute of Communication and Computer Systems, National Technical University of Athens, Athens, Greece

**Keywords:** Tumor growth, Cellular diffusion, Chemical diffusion, In silico modeling, Computational modeling

## Abstract

**Background:**

Contemporary biological observations have revealed a large variety of mechanisms acting during the expansion of a tumor. However, there are still many qualitative and quantitative aspects of the phenomenon that remain largely unknown. In this context, mathematical and computational modeling appears as an invaluable tool providing the means for conducting in silico experiments, which are cheaper and less tedious than real laboratory experiments.

**Results:**

This paper aims at developing an extensible and computationally efficient framework for in silico modeling of tumor growth in a 3-dimensional, inhomogeneous and time-varying chemical environment. The resulting model consists of a set of mathematically derived and algorithmically defined operators, each one addressing the effects of a particular biological mechanism on the state of the system. These operators may be extended or re-adjusted, in case a different set of starting assumptions or a different simulation scenario needs to be considered.

**Conclusion:**

In silico modeling provides an alternative means for testing hypotheses and simulating scenarios for which exact biological knowledge remains elusive. However, finer tuning of pertinent methods presupposes qualitative and quantitative enrichment of available biological evidence. Validation in a strict sense would further require comprehensive, case-specific simulations and detailed comparisons with biomedical observations.

## Background

### Introduction

Cancer is one of the main causes of mortality in the world. Statistics estimate that about one fifth of the population will suffer from cancer at some point in their lives [1]. Cancer is a category of diseases, which share several common features including sustained and uncontrolled cell proliferation, resistance to cell death, induction of angiogenesis, and activation of invasion and metastasis mechanisms [2].

The exact mechanisms that initiate cancer development remain largely unknown. However, it is widely accepted that cancer originates from cells which, due to various gene mutations, escape the body’s natural mechanisms of controlling the balance between cell proliferation and cell death [3]. These cells create a clump which grows faster than host cells. However, this small tumor grows with a decreasing rate; as the tumor grows, disorganization of the host vasculature and limited diffusion of nutrients to the center of the tumor lead to the formation of an internal necrotic core [4, 5]. Cells in the outer rim of the tumor proliferate, while cells in the interior die. For the tumor to grow large and become malignant, it needs to establish its own blood supply network, a process called angiogenesis. Angiogenesis results in a highly disorganized, tortuous and dilated vasculature, [4, 6–8] which however, provides the nutrients needed for further tumor growth. Evidently, during both the avascular and vascular phase of tumor development, the provision of nutrients to tumor cells through the blood supply network is highly inhomogeneous and time varying [4, 9–11]. In fact, many studies have shown that tumors contain hypoxic and hypoglycemic regions, particularly near the center, which affect local cell proliferation and death rates [4, 12, 13] and references therein.

Contemporary medical and biological literature on the subject shows that the vast majority of observations and results are conclusive only up to a point. In fact, there are still many qualitative and quantitative aspects of tumor progression that remain largely unknown. In this context, in silico modeling appears to be an invaluable tool for simulating scenarios and testing hypotheses pertaining to the aforementioned biological phenomena. To this end, this work describes an extensible and computationally efficient framework for in silico modeling of tumor growth in a 3-dimensional, inhomogeneous and time-varying chemical environment.

### Related literature

During the last few decades, mathematical and computational modeling of tumor growth has received a lot of attention from the scientific community. However, as noted in [14], there are no established first principle theories in cell-tissue modeling, and this seems to be the case even for today. Furthermore, there is still no generally agreed consensus on which modeling approach is the most suitable for modeling tumor growth. Scientists with different backgrounds have employed a variety of methods to attack the problem, in an effort to provide tools for conducting in silico experiments, which are significantly cheaper and less tedious than real laboratory experiments. Inspection of the literature shows that papers on the particular subject fall into two categories. Papers in the first category aim at an at least partial validation of a model against actual measurements. Such works include [15–18]. Papers in the second category aim at proposing new modeling methods or advancing already existing ones. Our work belongs in the second category.

In this section, we will review the main methods which have been most commonly employed in the pertinent literature.

Population models were probably among the first, simplest and yet effective of these approaches and utilize both deterministic and stochastic mathematics [19]. These models neglect tissue spatial structure and focus on the dynamics of the involved cell populations. They can address a variety of phenomena such as tumor clonal heterogeneity [20–22], tumor-host cell interactions [23–25] and response to therapy [26–29].

Despite the usefulness of population models, the spatial structure of tumors and the tissues they grow in appears to play an important role in tumor growth. To address this, several models have been proposed, with discrete entity, cell-based models forming a concrete class of such approaches. In these models, each tumor cell is treated as a discrete agent reacting to changes in its environment according to its own internal decision mechanism. Partial differential equations are most usually employed to model the background chemical environment. Most common approaches include lattice-based [30–42], lattice-free [14, 43–45] and Potts models [46–50]. Discrete agent models can address pertinent cellular, biochemical and biomechanical phenomena in considerable detail. However, they are computationally expensive and thus can simulate tumor sizes ranging in the order of at most 10^6^ cells, often considered in 2 dimensions.

Another popular approach is modeling the concentrations of both cells and chemical substances as continuous, spatially distributed quantities. Reaction-advection-diffusion equations are most commonly employed to model multicellular tumor spheroids [51–54]. Mainly for (but not limited to) the case of gliomas, the reaction diffusion equation is invoked to model the infiltration of tumor cells in the surrounding healthy tissue [55–61].

In [62, 63] the authors simulated the temporal evolution of non-necrotic, 2- and 3- dimensional tumors modeled as a continuum using a moving boundary. Using level set methods, this approach was extended in [64–68]. These papers demonstrate 2-dimensional simulations which additionally considered angiogenesis, necrosis and features of the tumor microenvironment.

A different approach, employing diffuse interface, multiphase mixture models was taken in [69, 70]. In these works, tissue is modeled as a multiphase mixture of solid components (e.g. dead tumor cells, viable tumor cells, host tissue) and water. Their temporal evolution is derived by mass equations and thermodynamic constitutive laws. Further work on this approach includes [71–73] where the authors consider additional phenomena like angiogenesis and biomechanical effects.

Miscellaneous approaches include the spatially averaged cellular automata developed in [74–76] where space is discretized in voxels, each one containing a number of cells. The resulting cubic grid is treated as a cellular automaton, with specified rules governing the transition of cells through the cell cycle phases within each voxel, the expansion of the tumor and the effects of various treatment modalities. In [77, 78] a hybrid approach was taken; cell distributions were modeled as continuous quantities except for the proliferating regions at the tumor boundary, where cells were treated as discrete entities. For a recent collection of articles on multiscale cancer modelling we refer to [79].

## Results

This paper develops a method for modeling 3-dimensional tumor growth with an emphasis on macroscopic variables. More specifically, we focus on variables quantifying the spatial distribution of cells and molecules, the provision of nutrients by the vascular system, tumor cell proliferation and invasion, the effects of tumor-induced vascular remodeling and how they affect each other as the tumor grows. Inspired by the aforementioned literature, our work aims at providing an additional useful tool for in silico experimentation with the following properties:

- The resulting model is modular; that is, it consists of several discrete mathematical/algorithmic modules, each one addressing a particular biological phenomenon. This allows keeping track of the assumptions made for each module. Furthermore, it facilitates model readjustment in case a new set of hypotheses needs to be considered. As we will show in the following sections, this can be done by extending or even completely redesigning the modules pertaining to the new hypotheses.
- Although some of the models mentioned in the literature can address phenomena even at the single cell level, they are in general computationally intensive. For some of them, simulation times in the order of 10–24 h have been reported [39, 69]. This imposes restrictions on the shape and size of the simulated tissue; therefore, many models consider only 2-dimensional tumors or spheroids with maximum size in the order of a few mm^3^. However, realistic tumors can grow up to several cm^3^ in volume. Besides that, as demonstrated in [40], results obtained from simulated small tissue areas generally cannot be extrapolated to larger domains. This implies that a balance between consideration of microscopic details and the ability of simulating larger regions of tissue must be kept. The methods developed in this paper aim at a resulting model that can simulate large (in the order of cm^3^) areas of tissue in 3 dimensions, with a spatial resolution in the order of 1–2 mm^3^, i.e. the voxel size of contemporary imaging techniques.
- Tumor growth consists in a complex interaction of phenomena evolving in different time scales. From the macroscopic point of view we adopt, the shortest time scale concerns the diffusion of molecules (seconds) and the largest one the overall tumor expansion (months). It is definitely a challenge to choose an appropriate simulation time step, i.e. one that addresses all the involved mechanisms in a sufficient amount of temporal detail, while keeping the simulation computationally tractable. Therefore, some models focus solely on cell proliferation and neglect the local availability and diffusion of nutrients [50, 54–61, 74–76]. Another common approach is to choose a time step in the order of minutes or hours, and solve the resulting (quasi-) steady state equations for the diffusion of nutrients [30–32, 41, 45, 52, 62–64, 66, 68, 70, 71, 73]. The methodology proposed in this work aims at time steps in the order of the shortest time scale, i.e. in the order of seconds.
- We also aim for computational efficiency, meant in a twofold sense.

- First, in conjunction with (b) and (c) above. The simulations we present here consider tissue areas in the order of 4.2 × 4.2 × 4.2 cm^3^, with a spatial resolution of 2 mm^3^ and a time step of 10 s for a time period of 3 months. The average simulation time on a standard desktop computer is about 10–12 min. The model is implemented in MATLAB; implementation in a precompiled language like C is expected to significantly decrease this time.
- Second, for scalability reasons, the resulting model should be able to exploit multicore computation. We will show in part VII of the methods section that the collection of all model variables at each time instant (i.e. the state vector) essentially evolves in a dynamical systems fashion. Given the current state, the next state can be calculated by a sequential application of algorithmically defined operators. Each one of these operators is perfectly eligible for parallelization, thereby enabling implementations considering larger tissue areas with finer spatial and temporal resolutions.

## Discussion

In this paper we present a novel methodology to approach the still open problem of modeling tumor growth. The presented modeling framework casts the problem in the realm of spatially distributed, stochastic dynamical systems by placing all pertinent spatial variables in a set of vectors, which collectively define the state vector of the overall system. At each time instant, a series of mathematically derived and algorithmically defined operators, each one corresponding to a particular biological mechanism, are applied to the state vector. Within the proposed framework, each one of these operators may be redesigned to consider different sets of starting assumptions, resulting generally in computationally efficient implementations. This facilitates the design of a large variety of hypothesis testing scenarios and corresponding in silico experiments, a process which, with the current limited qualitative and quantitative knowledge on the subject, seems inevitable. Several use cases are presented. Since we present simulations for a specific model, we do not attempt more detailed comparisons with biological data. Undoubtedly, there is still a lot of ground to be covered. Enrichment of both biological measurements and pertinent qualitative observations is necessary for further improvement of such methods. Future work should include much more case-specific and detailed comparisons between simulation results and available biological evidence.

## Conclusions

We have developed an extensible and computationally efficient framework for modeling tumor growth in a three-dimensional inhomogeneous and time-varying chemical environment, which constitutes an in silico alternative for testing different hypotheses and simulation scenarios. The model has been applied in the context of several use cases in order to visualize various aspects of tumour expansion and a multivariate analysis of the effects of model parameters on the number of live cancer cells of a growing tumor has been performed. Since many aspects of the pertinent biological mechanisms remain still largely unknown, finer tuning and validation of the simulation system in a strict sense presupposes qualitative and quantitative enrichment of the available biological evidence.

## Methods

The rest of the paper is organized as follows. In section I we present the main ideas used to model the diffusion of particles. In section II, we discuss boundary conditions. Sections III and IV specialize the ideas of the previous sections in the cases of chemical and cellular diffusion. Section V discusses tumor cell metabolism and consumption of nutrients, and how they affect proliferation and necrosis. In section VI we model the macroscopic effects of tumor-induced vascular remodeling. Section VII presents the complete model architecture. In section VIII we present some use cases, including a multivariate study on the effects of various model parameters on the number of viable tumor cells after a period of free growth.

### I. Modeling the diffusion of particles

The diffusion of particles is a natural phenomenon present in a vast variety of models regarding tumor growth. To model such phenomena, the diffusion partial differential equation is most commonly invoked:

1 $$ \frac{\partial c}{\partial t}=\nabla \cdot \left(D\nabla c\right) $$

where *c*(***x***, *t*) is the concentration of the species under consideration (cells or molecules) at time *t* and location ***x***, and *D* the diffusion tensor of the species in the surrounding material. This equation has been widely used to model cell diffusion, particularly in the case of glioblastoma, as well as diffusion of molecules in tissue. In the case of isotropic diffusion *D* is a constant scalar, and equation (1) reduces to

2 $$ \frac{\partial c}{\partial t}=D{\varDelta}_{\boldsymbol{x}}c $$

where *Δ*_***x***_ is the Laplace operator in *ℝ*^3^. In [80] we elaborated on the observation that (2) is the Fokker-Planck equation corresponding to the stochastic differential equation

3 $$ {d\boldsymbol{x}}_t=\sqrt{2D}\cdot {d\boldsymbol{B}}_t $$

where ***B***_*t*_ denotes the standard Brownian motion in *ℝ*^3^. Given the initial position ***x***_*o*_ of a particle, the distribution of the random variable ***x***_*t*_ (i.e. the solution of (3) at time *t*) provides *c* (***x***, *t*), i.e. the probability distribution over all possible locations of this particle at time *t*. The initial position of the particle may also be given in terms of a probability distribution *c* (***x***, 0). In this case, the probability distribution *c* (***x***, *t*) can be found by either of two equivalent ways: By solving (2) as a partial differential equation with initial value *c* (***x***, 0) to find the timely evolution of this distribution, or equivalently, by solving the stochastic differential equation (3) with initial distribution *c*(***x***, 0) to find the probability distribution of the random variable ***x***_*t*_.

To consider the collective movement of a population of particles, that is, molecules or cells located within a specified anatomic region, this notion of distribution can be utilized as follows: integration of *c*(***x***, *t*) over a region *A* of *ℝ*^3^ provides the fraction of the total particle population that is located in *A* at time *t*.

To model anisotropic diffusion, i.e. the preferential stochastic movement of particles along locally specific unit directions, the notion of the local diffusion ellipsoid (i.e. the diffusion tensor) is needed [59]. This notion corresponds to defining an ellipsoid in each point of the 3-dimensional space under consideration. Mathematically, this is made explicit by defining, in each point, a 3 × 3 positive definite symmetric matrix:

$$ D=\left[\begin{array}{ccc}{D}_{xx}& {D}_{xy}& {D}_{xz}\\ {}{D}_{xy}& {D}_{yy}& {D}_{yz}\\ {}{D}_{xz}& {D}_{yz}& {D}_{zz}\end{array}\right] $$

**Fig. 1:**
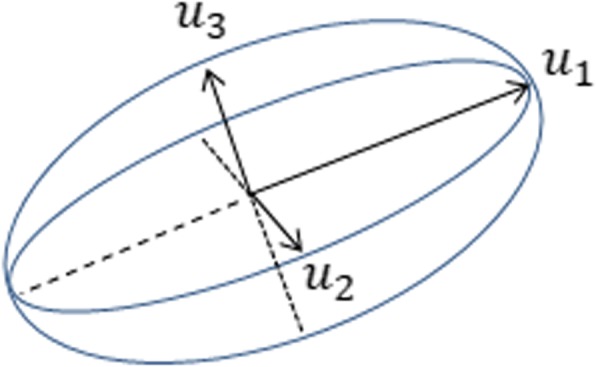
The diffusion ellipsoid

This matrix can be decomposed in the following form:

$$ D=\left[{u}_1\kern0.5em {u}_2\kern0.5em {u}_3\right]\left[\begin{array}{ccc}{\lambda}_1& 0& 0\\ {}0& {\lambda}_2& 0\\ {}0& 0& {\lambda}_3\end{array}\right]{\left[{u}_1\kern0.5em {u}_2\kern0.5em {u}_3\right]}^T $$

where *λ*_1_, *λ*_2_, *λ*_3_ are the eigenvalues of *D* and *u*_1,_*u*_2_, *u*_3_ are the corresponding orthonormal eigenvectors. We note that *D* is positive definite, hence *λ*_1_, *λ*_2_ and *λ*_3_ are positive numbers. The eigenvalues and eigenvectors of *D* define an ellipsoid whose principal axes lie on the directions of *u*_1_, *u*_2_ and *u*_3_. The principal axes have lengths $2\sqrt{\lambda_1}$, $2\sqrt{\lambda_2}$ and $2\sqrt{\lambda_3}$, respectively. The diffusion ellipsoid is depicted in Fig. 1.

The anisotropic diffusion of particles as dictated by the (local) diffusion ellipsoid can be modelled as follows; let *p*(***x***, ***x***_*o*_, *dt*) denote the probability of a particle starting at ***x***_*o*_ to be at ***x*** after time *dt*. Then

4 $$ p\left(\boldsymbol{x},{\boldsymbol{x}}_o, dt\right)=\frac{1}{{\left(2\pi \right)}^{\raisebox{1ex}{$3$}\!\left/ \!\raisebox{-1ex}{$2$}\right.}\det {\left(U{L}_a{U}^T\right)}^{\raisebox{1ex}{$1$}\!\left/ \!\raisebox{-1ex}{$2$}\right.}}\mathit{\exp}\left(-\frac{1}{2}{\left(\boldsymbol{x}-{\boldsymbol{x}}_o\right)}^TU{L}_a^{-1}{U}^T\left(\boldsymbol{x}-{\boldsymbol{x}}_o\right)\right) $$

Where

$$ {L}_a=\left[\begin{array}{ccc}{\lambda}_1\alpha & 0& 0\\ {}0& {\lambda}_2\alpha & 0\\ {}0& 0& {\lambda}_3\alpha \end{array}\right]\kern0.5em {L}_a^{-1}=\left[\begin{array}{ccc}\frac{1}{\lambda_1\alpha }& 0& 0\\ {}0& \frac{1}{\lambda_2\alpha }& 0\\ {}0& 0& \frac{1}{\lambda_3\alpha}\end{array}\right]\kern0.5em U=\left[\begin{array}{ccc}{u}_1& {u}_2& {u}_3\end{array}\right] $$

Note that the right-hand side of (4) is essentially an anisotropic Gaussian in *ℝ*^3^. The parameter *α* is a positive scalar, specific to the particles under consideration which in our case, will be tumor cells. This parameter rescales the eigenvalues, thereby rescaling conformally the axes of the diffusion ellipsoid. This reflects the fact that different kinds of particles may tend to move along the axes of same ellipsoid, but may do so with different velocities. Of note, since we are considering a local diffusion ellipsoid, in the most general case both eigenvalues and eigenvectors are functions of the position ***x***. After some mathematical elaborations detailed in [80], equation (4) leads us to model anisotropic diffusion by

$$ {\boldsymbol{x}}_{t+ dt}-{\boldsymbol{x}}_t=U\left[\begin{array}{ccc}\sqrt{\alpha {\lambda}_1}& 0& 0\\ {}0& \sqrt{\alpha {\lambda}_2}& 0\\ {}0& 0& \sqrt{{\alpha \lambda}_3}\end{array}\right]b $$

where *b* is a 3-dimensional, normally distributed random vector with zero mean and covariance matrix the identity matrix times *dt*:

$$ b\sim \frac{1}{{\left(2\pi \right)}^{\raisebox{1ex}{$3$}\!\left/ \!\raisebox{-1ex}{$2\ $}\right.}{(dt)}^{\raisebox{1ex}{$3$}\!\left/ \!\raisebox{-1ex}{$2$}\right.}}\mathit{\exp}\left(-\frac{b^Tb}{2 dt}\right) $$

$$ =\frac{1}{{\left(2\pi \right)}^{\raisebox{1ex}{$3$}\!\left/ \!\raisebox{-1ex}{$2\ $}\right.}{(dt)}^{\raisebox{1ex}{$3$}\!\left/ \!\raisebox{-1ex}{$2$}\right.}}\mathit{\exp}\left(-\frac{{\left\Vert b\right\Vert}^2}{2 dt}\right) $$

or equivalently, by the stochastic differential equation

5 $$ {d\boldsymbol{x}}_t=\sqrt{a}\cdot U\left(\boldsymbol{x}\right){L}^{\raisebox{1ex}{$1$}\!\left/ \!\raisebox{-1ex}{$2$}\right.}\left(\boldsymbol{x}\right){d\boldsymbol{B}}_t $$

For a given initial distribution *c*(***x***, 0) the solution of (5) provides the probability distribution of the random variable ***x***_*t*_, which can be interpreted exactly as described for the isotropic case. In the case of brain tumors, measurements concerning the diffusion tensor are obtained through the Diffusion Tensor Imaging (DTI) technique [56, 59].

In our approach, equations (3) and (5) will constitute the theoretical basis for modelling chemical and cellular diffusion.

Let us now assume that the diffusion of the particles (molecules or cells) under consideration takes place in a cubic lattice consisting of *N* × *N* × *N* geometrical cells (voxels). Each voxel is a cube of dimensions *Δs* × *Δs* × *Δs*. We fix a temporal discretization step equal to *Δτ*. Voxels in that cubic lattice can be classified into 4 categories, depending on the number of their neighboring voxels within the lattice. Voxels in the interior of the lattice have 26 neighbors. Voxels at the outer faces of the lattice have 17 neighbors. Voxels at the outer edges of the lattice have 11 neighbors and voxels at the outer vertices of the lattice have 7 neighbors. Furthermore, for each particular voxel, its neighboring voxels fall into 3 categories: the ones that share a common face, the ones that share a common edge and the ones that share a common vertex with the particular voxel.

For the remaining part of this section, let us adopt the assumption that, at each discrete time point, the distribution of the particles under consideration within each voxel is uniform. This is somewhat oversimplifying, and we will further elaborate on this assumption in the following sections, where we specifically consider chemical or cellular diffusion. For the moment, this assumption will render the presentation of the main ideas more straightforward.

Under the uniformity assumption, at any discrete time point *t* and for any pair of voxels *A* and *B* (not necessarily different) we can calculate the probability for a particle to lie within *B* at time *t* + *Δτ*, *given* that its position at time *t* is a uniformly distributed (u.d.) random variable supported in *A*. Numerical integration of equations (3) and (5) for a uniform initial distribution provides the means for a Monte Carlo calculation of this probability, which we will denote by *Pr*(*A* → *B*).

Let *A* denote a voxel not lying on the boundary of the lattice nor being adjacent to it, and *B*_*i*_, *i* = 1, …, 26 its neighbors. A key step to our discretization process is to choose the voxels’ edge length *Δs* and the time step *Δτ* such that, from one time instant to the next one, the particles lying in each voxel diffuse at most into its neighbors. Mathematically, this means that *Δs* and *Δτ* should be chosen such that

6 $$ \mathit{\Pr}\left(A\to A\right)+{\sum}_{i=1}^{26}\Pr \left(A\to {B}_i\right)=1 $$

If (6) holds then, for a spatially constant, isotropic diffusion like the one implied in (3), for each voxel *A* not lying on the boundary of the lattice nor being adjacent to it, these probabilities consist of essentially 4 numbers: one for the particles that are in *A* and will remain in *A* (i.e. *Pr*(*A* → *A*)) and three more, one for each of the common face, common edge and common vertice neighbors of *A*. Furthermore, for any two voxels *A* and *B* it holds that *Pr*(*A* → *B*) =  *Pr* (*B* → *A*). For a spatially varying, anisotropic diffusion like the one implied in (5), these symmetries do not hold; the respective probabilities should be precalculated independently for any particular voxel.

Thus, under the uniformity assumption, for any voxel *A* not lying on the boundary of the lattice nor being adjacent to it, knowing the particle population of the voxel *Q*_*t*_(*A*) and its neighbors *Q*_*t*_(*B*_*i*_), *i* = 1, …, 26 at a time instant *t*, allows us to calculate the population within A at the next time instant *t* + *Δτ* by

7 $$ {Q}_{t+\varDelta \tau}(A)=\mathit{\Pr}\left(A\to A\right){Q}_t(A)+{\sum}_{i=1}^{26}\mathit{\Pr}\left({B}_i\to A\right){Q}_t\left({B}_i\right) $$

Apparently, this equation does not hold as such when the voxel under consideration lies on the boundary of the lattice. We will deal with these voxels (and their neighbors) in detail in the next section, where we discuss boundary conditions.

In what follows, it will come in handy to represent particle quantities within the voxels in vector form. Let us make this representation explicit by the following construction. Let the coordinates of each voxel in the lattice be given by a triad of integers, (*i*, *j*, *k*) where *i*, *j*, *k* = 1, …, *N*. We define a mapping *L* : *ℕ*^3^ → *ℕ* as follows: *L*(*i*, *j*, *k*) = *i* + (*j* − 1)*N* + (*k* − 1)*N*^2^. This mapping is a bijection from the set of triads (*i*, *j*, *k*), *i*, *j*, *k* = 1, …, *N* to the integers from 1 to *N*^3^. Let *Q*(*i*, *j*, *k*) denote the quantity of particles in the respective voxel. We define the vector *q* of *N*^3^ elements by *q*(*L*(*i*, *j*, *k*)) = *Q*(*i*, *j*, *k*). By this construction, the elements of the 3d matrix *Q* are explicitly mapped to the elements of the one-dimensional vector *q*.

### II. Dirichlet and Neumann boundary conditions

Knowing the particle population within each voxel of the lattice at a time instant, equation (7) allows us to calculate the population within each voxel at the next time instant for all the voxels, except the ones on the boundary of the lattice and their neighbors. Each of the boundary voxels has less than 26 neighbors. In fact, each of these voxels would have 26 neighbors in an infinite lattice, but not all of them are included in a bounded, *N* × *N* × *N* lattice. Unless we specify boundary conditions, the calculation in (7) cannot be carried out neither for those voxels and consequently, nor their neighbors. There are two types of boundary conditions that can be imposed in a classical diffusion problem, those of the Dirichlet type and those of the Neumann type. In this work, we consider two specific types of such boundary conditions, namely, the time-independent Dirichlet and the homogeneous Neumann boundary conditions.

Time-independent Dirichlet boundary conditions express the requirement that on the boundary of the region under consideration, the quantity of interest does not change with time. In the framework presented here, this is expressed mathematically by the following: If a voxel *A* lies on the boundary of the lattice, to calculate its population at the next time instant, instead of equation (7) simply apply *Q*_*t* + *Δτ*_(*A*) = *Q*_*t*_(*A*). Additionally, if a voxel is adjacent to the boundary, simply apply (7) by using the respective probabilities as they are calculated from the numerical integration of (3) or (5).

We note that in the case of tumor growth, some authors have also considered time-dependent, periodic Dirichlet boundary conditions [40]. This is also feasible in the proposed framework, and can be implemented as follows. For any boundary voxel *A*, at any time instant *t*, apply *Q*_*t*_(*A*) = *g*_*t*_, where *g*_*t*_ is the desired periodic function.

Homogeneous Neumann boundary conditions express the requirement that the flux of the quantity of interest across the boundary of the region under consideration should be zero, i.e. the boundary is non-permeable. In terms of calculus this is expressed by the requirement, at any time instant and at any point of the boundary, the projection of the gradient of the quantity on the outward normal of the boundary at that point to be zero. In the stochastics literature, a non-permeable boundary within which a random motion takes place is often referred to as a reflecting boundary [81]. In the framework presented here, this is expressed mathematically as follows.

As previously mentioned, any voxel *A* lying on the boundary has 17, 11 or 7 neighbor voxels which we denote by *B*_*i*_, where *i* is an integer from 1 up to 17, 11 or 7, depending on the position of the voxel. For each such voxel *A*, and its neighbors *B*_*i*_ we calculate the probabilities Pr(*A* → *B*_*i*_) as described in the previous section, but only for the voxels that are contained in the lattice. Specifically, if *A* lies on the boundary, we calculate the probabilities Pr(*A* → *A*), Pr(*A* → *B*_*i*_) where *i* is an integer from 1 up to 17, 11 or 7. We then normalize these probabilities to sum to one. By this calculation we acquire the probabilities we need, in order to apply equation (7) for any voxel in the lattice. Using these normalized probabilities when applying equation (7) for either boundary voxels, or their neighbors, ensures that every particle lying in a voxel on the boundary, will remain within the lattice, that is, within the region of interest, thereby capturing the notion of a reflecting boundary.

In our case, the region of interest is a cube. It is apparent that these methods of imposing Dirichlet or Neumann boundary conditions apply to more complex shapes, as long as space is properly discretized [82]. The case of brain tumors, where the skull naturally imposes a reflecting boundary to the diffusion of tumor cells is an example where this approach may be useful.

### III. Diffusion of glucose and oxygen

In this section we will use the ideas presented previously to develop a model for the diffusion of chemical molecules (glucose or oxygen) in the region of interest, that is, the cubic lattice of dimensions *N* × *N* × *N*. For the moment, we will assume that the diffusion is isotropic and that the distribution of molecules within each voxel is uniform. Let *q*_*t*_ denote the *N*^3^ × 1 vector whose entries are the quantities of the molecule under consideration within each voxel at time *t*. Αssuming time-independent Dirichlet boundary conditions, equation (7) implies that if we know *q*_*t*_, we can calculate *q*_*t* + *Δτ*_ by performing a linear calculation. This means that there is a *N*^3^ × *N*^3^ square matrix *T* such that *q*_*t* + *Δτ*_ = *Tq*_*t*_, .

We remind the reader that due to the symmetries holding for isotropic diffusion, for each voxel *A* not on the boundary, we can apply equation (7) by using only four numbers. We denote these numbers by Pr(*A* → *A*), Pr(*F* → *A*) (for common face neighbors), Pr(*E* → *A*) (for common edge neighbors), and Pr(*V* → *A*) (for common vertex neighbors). These numbers can be precalculated by numerically integrating (3) for a uniform initial distribution, where in each case, *D* is taken to be the diffusion coefficient of the respective molecule. We subsequently use these values and the mapping *L* defined in section I to construct the matrix *T* according to the following algorithm:

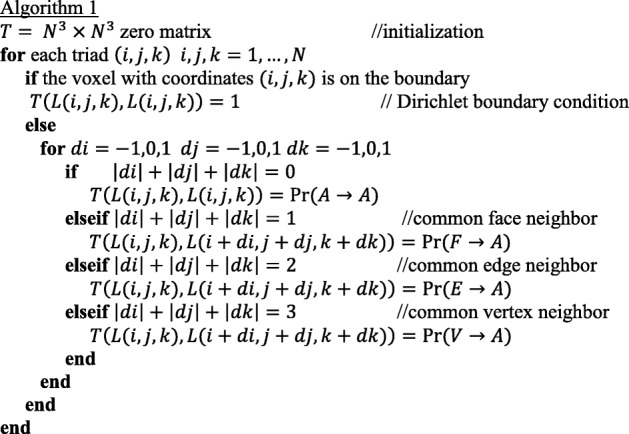



Note that, in view of the mapping *L*, each row *i* of *T* corresponds to a specific voxel *A*, i.e. to a specific position *i* of the vector *q*_*t* + *Δτ*_. The nonzero entries of the particular row correspond to the probabilities Pr(*A* → *A*) and Pr(*B*_*i*_ → *A*), where *B*_*i*_ are the neighbors of *A*, as indicated by (7). Furthermore, each column *j* of *T* corresponds to a specific voxel *B*, i.e. to a specific position *j* of the vector *q*_*t*_. The nonzero entries of the particular column correspond to the probabilities Pr(*B* → *B*) and Pr(*B* → *A*_*i*_), where *A*_*i*_ are the neighbors of *B*. Probabilities of the type Pr(*A* → *A*) lie in the main diagonal of *T*.

In our model, we will use two such matrices, one for glucose and one for oxygen, denoted by *T*_*gl*_, *T*_*o*_ respectively. Each of the respective *N*^3^ × 1 vectors will be denoted by *gl*_*t*_, *o*_*t*_.

The matrix constructed by Algorithm 1 has an interesting property. For sufficiently large *N* and due to the assumption implied in equation (6), it is a sparse matrix: in each row, at most 27 elements are nonzero. This provides a significant relief of the computational burden of the entire model.

Note that all arguments in this section, resulting in the simple model *q*_*t* + *Δτ*_ = *Tq*_*t*_ for molecular diffusion, rely on the uniformity assumption as it was stated in section I.

We mentioned that this assumption is somewhat oversimplifying. Indeed, in vivo measurements in tumor areas show that chemical gradients can be spatially non-uniform and time varying. Reportedly, oxygen profiles can vary locally up to 50% within an hourly time frame [83]. The highly irregular tumor vasculature may further complicate things and pertinent biological mechanisms remain largely unknown [6, 7]. Inherent stochasticity is also expected to play a role. Consequently, detailed quantification of the inflicted macroscopic effects such as time evolution of chemical fields seems currently infeasible. Therefore, we broaden our perspective as follows.

First, we note that our calculations showed that for both oxygen and glucose, it holds that Pr(*A* → *A*)> Pr(*F* → *A*)> Pr(*E* → *A*)> Pr(*V* → *A*) and that each of these numbers is one order of magnitude greater than the next one. Therefore, we will use the precalculated numbers Pr(*A* → *A*), Pr(*F* → *A*), Pr(*E* → *A*), and Pr(*V* → *A*) only as estimates for the (relative) orders of magnitude of these probabilities. To take the largely stochastic, collective effect of the aforementioned complex mechanisms and uncertainties into consideration, we will choose a time step *Δτ* in the order of seconds (i.e. the time scale of chemical diffusion) and introduce a certain degree of randomness to the matrices *T*_*gl*_ and *T*_*o*_. Specifically, every some time steps, each column of these matrices corresponding to a voxel in the interior or in the vicinity of the tumor will be randomly perturbed, such that its nonzero entries retain their relative orders of magnitude and their sum remains one. We provide the respective implementation details in the appendix.

Introducing stochasticity implies that for each simulation scenario, several simulations will be required. Nonetheless, the results of these multiple simulations will enable a more comprehensive consideration of possible outcomes.

### IV. Diffusion of tumor cells

To model the diffusion of cancer cells, we make the following assumptions:

i. There are four types of cells within each voxel; live normal (host) cells, necrotic normal cells, live tumor cells and necrotic tumor cells.
ii. For any voxel, there is an average cell population capacity (tumor +normal) which we denote by *M* and a maximum cell population capacity which we denote by *M*_*max*_.
iii. As the tumor grows, live normal cells become either dislocated by invading tumor cells or necrotic.
iv. When the sum of living cancer, necrotic cancer and necrotic host cells in a voxel exceeds *M*_*max*_, living cancer cells in excess of *M*_*max*_ invade neighboring voxels according to equation (5).
v. Both tumor and normal necrotic cells remain in the voxel they became necrotic, that is, they do not “invade” neighboring voxels.

Our model will be based on equations (5) and (7). Since we assume that in each voxel, only tumor cells in excess of *M*_*max*_ diffuse to neighboring voxels, the probability *Pr*(*A* → *A*) is not needed. For each voxel *A* and its neighbors *B*_*i*_ we calculate the probabilities Pr(*A* → *B*_*i*_) and normalize them to sum to 1. We then place these probabilities in the respective rows and columns of a matrix *T*_*c*_, similar to the matrices *T*_*gl*_ and *T*_*o*_ of the previous section. Of note, the main diagonal of the matrix *T*_*c*_ consists of zeros, and not of probabilities *Pr*(*A* → *A*), as is the case with *T*_*gl*_ and *T*_*o*_. Apparently, the matrix *T*_*c*_ is also sparse, lightening thereby the computational burden. Furthermore, by construction, the matrix *T*_*c*_ implies homogeneous Neumann boundary conditions for living tumor cells.

The tumor cell diffusion algorithm has a quite simple implementation. Let *l*_*t*_, *nc*_*t*_, *nn*_*t*_ denote the *N*^3^ × 1 vectors whose entries are respectively the live tumor, necrotic tumor and necrotic normal cells within each voxel at time *t* (we use this notation throughout the text, please see also the first paragraphs of the following section). Let *w*_*t*_ denote the sum of these vectors. Let $\overline{M_{max}}$ denote the *N*^3^ × 1 vector whose entries are all *M*_*max*_. We also adopt the following notation: for a vector *x* and a number *μ*, the vector (*x* ≥ *μ*) is the binary vector with elements set to 1 if the corresponding element of *x* is ≥*μ* and 0 otherwise. Finally, for any two vectors *x* and *y* let *x*. ∗ *y* denote their element-wise product. The resulting algorithm boils down to a simple vector algebraic representation:

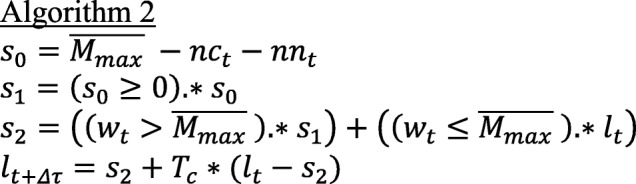



Vector *s*_1_ contains, for each voxel, the total number of live (tumor and normal) cells that the particular voxel can hold additional to its necrotic cells. In view of assumptions (ii), (iv) and (v), *s*_2_ entries are numbers of live cancer cells which already exist in each voxel that can also remain in the respective voxel. In view of assumptions (iii) and (iv), the vector (*l*_*t*_ − *s*_2_) contains the numbers of live tumor cells that lie within each respective voxel in excess of *M*_*max*_, and therefore invade neighboring voxels by dislocating live normal cells. The populations of live tumor cells at the next time instant, i.e. after their diffusion to neighboring voxels is given by the sum of *s*_2_ and *T*_*c*_ ∗ (*l*_*t*_ − *s*_2_).

Of note, the entire approach again relies on the uniformity assumption, but this time for tumor cells. Apparently, this is a simple, and definitely not the only way to model the invasion of tumor cells. For the simulations presented below, we will rely on it. More sophisticated approaches could introduce randomness in the matrix *T*_*c*_ exactly as described in section III; probabilities in *T*_*c*_ could be dynamically readjusted by taking into account nutrient quantities within each voxel, thereby introducing some at least rough, phenomenological notion of chemotaxis in the model. However, our aim for the moment is to keep the presentation of the main ideas as simple as possible. We will provide some suggestions for further elaboration and implementation of this module in the appendix.

### V. Proliferation/necrosis according to cell metabolism and local availability of oxygen and glucose

In this section, we will study the proliferation of cells within a voxel *A*. For a time interval equal to the time step *Δτ*, we will neglect diffusion phenomena, and study the proliferation and necrosis of cells within *A* as they are dictated by the cells’ consumption needs and the local availability of oxygen and glucose. Although special effort has been made in order for the proposed approach to be founded on a consensus of biological evidence, it is definitely not the only one possible; somewhat different assumptions may lead to different approaches. In section VII, where we present the complete model architecture, it will become apparent that the approach adopted here can be completely readjusted, in order to consider different assumptions.

For any voxel *A*, we will need the following variables:

- *l*_*t*_(*A*): number of living cancer cells within *A* at time *t*.

- *nc*_*t*_(*A*): number of necrotic cancer cells within *A* at time *t*.

- *nn*_*t*_(*A*): number of necrotic normal cells within *A* at time *t*.

The number of live normal cells within *A* at time *t* is given by *s*(*M* − *l*_*t*_(*A*) − *nc*_*t*_(*A*) − *nn*_*t*_(*A*)) where *s*(∙) is the known function *s*(*x*) = *x* if *x* ≥ 0 and 0 if *x* < 0.

- *o*_*t*_(*A*): Oxygen quantity (pmols) within *A* at time *t*. Initial value *o*_0_(*A*) is the same for every voxel, denoted by $\overline{o_0}$. $\overline{o_0}$ is calculated such that the initial concentration of oxygen in each voxel equals the concentration of dissolved oxygen in the blood (see section VI).

- *gl*_*t*_(*A*): Glucose quantity (pmols) within *A* at time *t*. . Initial value *gl*_0_(*A*) is the same for every voxel, denoted by $\overline{gl_0}$. $\overline{gl_0}$ is calculated such that the initial concentration of glucose in each voxel equals the concentration of glucose in the blood (see section VI).

- *o*_*b*_*t*_(*A*): Oxygen supply rate (pmols/sec) by the local vascular network within *A* *during* the previous time interval *t* − Δτ → *t*

- *gl*_*b*_*t*_(*A*): Glucose supply rate (pmols/sec) by the local vascular network within *A* *during* the previous time interval *t* − Δτ → *t*

The variables *o*_*b*_*t*_(*A*) and *gl*_*b*_*t*_(*A*) quantify macroscopically the role of the local vascular network in the provision of oxygen and glucose within each voxel. We will assume that their values remain constant *during* each time interval *t* → *t* + *Δτ*. In fact, these variables are too subject to a dynamic time evolution due to the effects of local vascular remodeling induced by the tumor. We will discuss this matter in detail in the next section. In this section, we will focus on the time interval *t* → *t* + *Δτ*, and aim at calculating the aforementioned cell populations and chemical quantities at the next time instant, i.e. *t* + *Δτ*. We note that the adopted notation implies that *o*_*b*_*t* + *Δτ*_(*A*), *gl*_*b*_*t* + *Δτ*_(*A*) denote the oxygen and glucose supply rates during the time interval under consideration, i.e. *t* → *t* + *Δτ*. In this section, these quantities are assumed to be (pre)calculated by the algorithm described in the next section.

We will further need the following parameters:

- *M*: average cell population capacity for each voxel.

- *K*_*o*_: oxygen consumption rate (pmols/sec) of a normal cell.

- *K*_*gl*_: glucose consumption rate (pmols/sec) of a normal cell. Typically, for a normal cell acquiring its energy mainly through combustion of glucose, *K*_*o*_ is 4 to 6 times larger than *K*_*gl*_ [36, 37, 84].

- *K*_*ATP*_: ATP consumption rate (pmols/sec) of a normal cell.

- *λ*: The product *λK*_*ATP*_ defines the ATP consumption rate of an actively proliferating tumor cell. Since proliferating tumor cells consume much more resources than normal cells, *λ* should be >1 [39, 46, 85]. For quiescent tumor cells, *λ* is assumed to be 1.

- *cc*: Cell cycle duration (sec).

- *a*_*max*_: maximum mitosis rate that tumor cells can achieve, when they are not limited by local oxygen and glucose levels. (mitoses/cell/ time step in secs).

Of note, concerning the initial values of *o*_*b*_*t*_(*A*) and *gl*_*b*_*t*_(*A*) i.e. *o*_*b*_0_(*A*) and *gl*_*b*_0_(*A*), we will make the following assumption. For every voxel at time *t* = 0 the provision of oxygen and glucose by the local vascular network and their consumption by normal cells should balance each other, such that the respective background concentrations remain constant, i.e. *o*_*b*_0_(*A*) = *MK*_*o*_ and *gl*_*b*_0_(*A*) = *MK*_*gl*_ [69, 71].

A normal cell’s ATP consumption is dictated by *K*_*o*_, *K*_*gl*_ and the stoichiometry of clean combustion and glycolysis:

$$ {\displaystyle \begin{array}{cc}\ \mathrm{clean}\ \mathrm{combustion}:& Gl\kern0.5em +\kern0.75em 6{O}_{2\kern0.5em }\to \kern0.75em 36\  ATP\ \\ {}& \left(\raisebox{1ex}{$1$}\!\left/ \!\raisebox{-1ex}{$6$}\right.\right){K}_o+{K}_o\to 6{K}_o\\ {} glycolysis:& Gl\to 2\  ATP\\ {}& {K}_{gl}-\left(\raisebox{1ex}{$1$}\!\left/ \!\raisebox{-1ex}{$6$}\right.\right){K}_o\to 2{K}_{gl}-\left(\raisebox{1ex}{$1$}\!\left/ \!\raisebox{-1ex}{$3$}\right.\right){K}_o\end{array}} $$

Thus, knowing *K*_*o*_, *K*_*gl*_ allows us to explicitly determine *K*_*ATP*_= $$ \left(\raisebox{1ex}{$17$}\!\left/ \!\raisebox{-1ex}{$3$}\right.\right) $$ *K*_*o*_+ 2*K*_*gl*_.

The stoichiometry of the clean combustion of glucose requires that the glucose/oxygen uptake ratio is 1:6. It is well documented that for cancer cells, due to increased utilization of glycolysis, this is not the case [2]. Experimental measurements and estimations report that the ratio of glucose /oxygen consumption in tumors can vary up to 1:1 or even more [14, 46, 86–88]. Compared to clean combustion, glycolysis is 18 times less efficient in ATP production and cancer cells compensate this deficiency by upregulating glucose transporters, thereby increasing glucose import in the cytoplasm. Considerably increased glucose uptake and utilization has been reported in a variety of tumors by the use of positron emission tomography (PET) [2]. It has been also reported that local levels of oxygen and glucose have an effect on this ratio [86, 89]. Quantitative details of these phenomena are still unclear. The possibility that a single cell may employ both glycolysis and normal aerobic metabolism is not excluded. Qualitatively it seems evident that when oxygen falls below a certain threshold, cells tend to switch to a glycolytic phenotype. However, this observation does not tell the whole story, since cancer cells switch to glycolysis even when oxygen levels are abundant [90, 91].

To take account of this evidence from a modeling perspective, we take the following approach. We assume that tumor cells within a voxel may obtain the energy they need (i.e. *λK*_*ATP*_ pmols/sec) by acquiring a fraction *β* of it by glycolysis and the remaining fraction by combustion. This fraction will depend on the local availability of oxygen and glucose, plus, we will introduce a degree of randomness in it. Two additional, case-specific parameters are the minimum and maximum values of this fraction, which we respectively denote by *β*_1_ and *β*_2_. Apparently, 0 ≤ *β*_1_ ≤ *β* ≤ *β*_2_ ≤ 1.

We have assumed that a quiescent tumor cell needs *K*_*ATP*_ pmols ATP/sec to stay alive. From this amount and according to the stoichiometry, *βK*_*ATP*_ pmols/sec should come from glycolysis of *βK*_*ATP*_/2 pmols/sec glucose. The remaining (1 − *β*)*K*_*ATP*_ pmols/sec should come from combustion of (1 − *β*)*K*_*ATP*_/36 pmols/sec glucose with (1 − *β*)*K*_*ATP*_/6 pmols/sec oxygen. Thus, to stay alive, a quiescent tumor cell needs

8 $$ {\displaystyle \begin{array}{c}\bullet \frac{17\beta +1}{36}{K}_{ATP}\kern0.28em \mathrm{pmol}\ \mathrm{glucose}/\sec\ \mathrm{and}\\ {}\bullet \frac{1-\beta }{6}{K}_{ATP}\kern0.28em \mathrm{pmol}\ \mathrm{oxygen}/\sec .\end{array}} $$

On the other hand, an actively proliferating tumor cell needs *λK*_*ATP*_ pmols ATP/sec. From this amount, *βλK*_*ATP*_ pmols/sec should come from glycolysis of *βλK*_*ATP*_/2 pmols/sec glucose. The remaining (1 − *β*)*λK*_*ATP*_ pmols/sec should come from combustion of (1 − *β*)*λK*_*ATP*_/36 pmols/sec glucose with (1 − *β*)*λK*_*ATP*_/6 pmols/sec oxygen. Thus, an actively proliferating tumor cell needs

9 $$ {\displaystyle \begin{array}{c}\bullet \frac{17\beta +1}{36}\lambda {K}_{ATP}\kern0.28em \mathrm{pmol}\ \mathrm{glucose}/\sec\ \mathrm{and}\\ {}\bullet \frac{1-\beta }{6}\lambda {K}_{ATP}\kern0.28em \mathrm{pmol}\ \mathrm{oxygen}/\sec .\end{array}} $$

With the above evidence and assumptions in mind, we devise the following algorithm, which will be executed for each voxel *A* at each time step. The involved calculations require a detailed analysis, consisting of several steps and subcases.

Step 1. In this step, we will calculate the following quantities:

*O*_*av*_: available oxygen for tumor cells in *A* during *Δτ*.

*Gl*_*av*_: available glucose for tumor cells in *A* during *Δτ*.

*nn*_*t* + *Δτ*_(*A*): number of necrotic normal cells in *A* at the next time instant.

First, we calculate the amount of oxygen and glucose that will be available for tumor cells, by subtracting the consumption of normal cells:

$$ {O}_1={o}_t(A)+o\_{b}_{t+\varDelta \tau}(A)\ \varDelta \tau -s\left(M-{l}_t(A)-{nc}_t(A)-{nn}_t(A)\right){K}_o\varDelta \tau $$

$$ {Gl}_1={gl}_t(A)+ gl\_{b}_{t+\varDelta \tau}(A)\ \varDelta \tau -s\left(M-{l}_t(A)-{nc}_t(A)-{nn}_t(A)\right){K}_{gl}\varDelta \tau $$

Case 1.1: *O*_1_ ≥ 0 and *Gl*_1_ ≥ 0. In this case, oxygen and glucose suffice for all normal cells in *A* to stay alive, hence,

$$ {O}_{av}={O}_1 $$

$$ {Gl}_{av}={Gl}_1 $$

$$ {nn}_{t+\varDelta \tau}(A)={nn}_t(A) $$

Case 1.2: *O*_1_ < 0 or *Gl*_1_ < 0. This means that either oxygen and/or glucose do not suffice for all normal cells in *A* to stay alive. We calculate

${N}_n=\mathit{\min}\left(\frac{o_t(A)+o\_{b}_{t+\varDelta \tau}(A)\varDelta \tau}{K_o\varDelta \tau},\frac{gl_t(A)+ gl\_{b}_{t+\varDelta \tau}(A)\varDelta \tau}{K_{gl}\varDelta \tau}\right)$, i.e. how many living normal cells in *A* will stay alive.

${\overline{N}}_n=s\left(M-{l}_t(A)-{nc}_t(A)-{nn}_t(A)\right)-{N}_n$, i.e. how many normal cells in *A* will become necrotic.

In this case, the aforementioned quantities *O*_*av*_, *Gl*_*av*_ and *nn*_*t* + *Δτ*_(*A*) are

$$ {\displaystyle \begin{array}{c}{O}_{av}={o}_t(A)+o\_{b}_{t+\Delta \tau }(A)\Delta \tau -{N}_n{K}_o\Delta \tau \\ {}{Gl}_{av}={gl}_t(A)+ gl\_{b}_{t+\Delta \tau }(A)\ \Delta \tau -{N}_n{K}_{gl}\Delta \tau \\ {}{nn}_{t+\Delta \tau }(A)={nn}_t(A)+{\overline{N}}_n\end{array}} $$

Step 2. In this step, by taking into account the results of Step 1, we will study the proliferation/necrosis of tumor cells. Eventually, we will calculate the quantities *l*_*t* + *Δτ*_(*A*), *nc*_*t* + *Δτ*_(*A*), *o*_*t* + *Δτ*_(*A*), *gl*_*t* + *Δτ*_(*A*). The quantity *nn*_*t* + *Δτ*_(*A*) has been calculated in step 1.

Case 2.1: If *l*_*t*_(*A*) = 0, i.e. there are no living tumor cells in the voxel, the calculation is simple:

$$ {l}_{t+\varDelta \tau}(A)={l}_t(A) $$

$$ {nc}_{t+\varDelta \tau}(A)={nc}_t(A) $$

$$ {o}_{t+\varDelta \tau}(A)={O}_{av} $$

$$ {gl}_{t+\varDelta \tau}(A)={Gl}_{av} $$

Case 2.2. If *l*_*t*_(*A*) > 0 the calculation is more elaborate. Let *a* be the mitosis rate of tumor cells per time step *Δτ*, i.e. the fraction of tumor cells within *A* that will divide during *Δτ* and *cc* the duration of their cell cycle. The duration of their cell cycle in time steps is *cc*/*Δτ*. Assuming a uniform distribution of proliferating cells at all time steps of the cell cycle, we estimate a total number *al*_*t*_(*A*)(*cc*/*Δτ*) of actively proliferating tumor cells. Some stochasticity may be introduced in this estimate, but to keep the presentation simple we will not go into details.

In view of (8) and (9), we define

$$ {O}_c={l}_t(A)\frac{1-\beta }{6}{K}_{ATP}\varDelta \tau +\left(\lambda -1\right){al}_t(A)\left( cc/\varDelta \tau \right)\frac{1-\beta }{6}{K}_{ATP}\varDelta \tau $$

$$ {Gl}_c={l}_t(A)\frac{17\beta +1}{36}{K}_{ATP}\varDelta \tau +\left(\lambda -1\right){al}_t(A)\left( cc/\varDelta \tau \right)\frac{17\beta +1}{36}{K}_{ATP}\varDelta \tau $$

The quantities *O*_*c*_ and *Gl*_*c*_ are the amounts of oxygen and glucose that will be needed by tumor cells in *A* in order to proliferate with mitosis rate *a*. Those quantities should be limited respectively by *O*_*av*_ and *Gl*_*av*_, calculated from Step 1. Mathematically, this is expressed by the inequalities *O*_*c*_ ≤ *O*_*av*_ and *Gl*_*c*_ ≤ *Gl*_*av*_. These inequalities require a closer examination and can be equivalently written in the form

10 $$ a\le \frac{O_{av}-{l}_t(A)\frac{1-\beta }{6}{K}_{ATP}\varDelta \tau}{\left(\lambda -1\right){al}_t(A)\left( cc/\varDelta \tau \right)\frac{1-\beta }{6}{K}_{ATP}\varDelta \tau} $$

11 $$ a\le \frac{Gl_{av}-{l}_t(A)\frac{17\beta +1}{36}{K}_{ATP}\varDelta \tau}{\left(\lambda -1\right){al}_t(A)\left( cc/\varDelta \tau \right)\frac{17\beta +1}{36}{K}_{ATP}\varDelta \tau} $$

The inequalities (10) and (11) involve the -up to now undetermined- variables *a* and *β*. They reflect the fact that, for the tumor cells in *A* to proliferate with mitosis rate *a*, a number *β* ∈ [*β*_1_, *β*_2_] should exist, such that *a* ≥ 0, and (10), (11) are satisfied.

Investigation of (10): For each *β* ∈ [*β*_1_, *β*_2_] we define the function

$$ {a}_o\left(\beta \right)=\frac{O_{av}-{l}_t(A)\frac{1-\beta }{6}{K}_{ATP}\varDelta \tau}{\left(\lambda -1\right){l}_t(A)\left( cc/\varDelta \tau \right)\frac{1-\beta }{6}{K}_{ATP}\varDelta \tau} $$

For each given *β* ∈ [*β*_1_, *β*_2_], we observe the following:

10a) If *a*_*o*_(*β*) ≥ 0: *a*_*o*_(*β*) is the maximum mitosis rate that the tumor cells can achieve for the specific *β*, subject solely to the limitations imposed by the available oxygen in *A*.

10b) If *a*_*o*_(*β*) < 0, it is implied that ${O}_{av}-{l}_t(A)\frac{1-\beta }{6}{K}_{ATP}\varDelta \tau <0.$ This means that for the specific *β*, no positive mitosis rate can be achieved. In fact, available oxygen does not suffice for all tumor cells in *A* to stay alive.

Furthermore:

10c) *β* = 1 means that tumor cells can acquire the energy they need relying solely on glycolysis. Note that *β* → 1 implies *a*_*o*_(*β*) →  + ∞, reflecting the fact that in this case, the proliferation of tumor cells is not limited by the available oxygen.

10d) For each *β* ∈ [0, 1), *a*_*o*_(*β*) is an increasing function of *β*.

10e) If a *β* ∈ [*β*_1_, *β*_2_] such that *a*_*o*_(*β*) ≥ 0 exists, it should also satisfy

$$ \beta \ge 1-\frac{6{O}_{av}}{l_t(A){K}_{ATP}\varDelta \tau}\equiv \underset{\_}{\beta } $$

Since *a*_*o*_(*β*) is increasing, $\underset{\_}{\beta }$ is actually the lowest number for which *a*_*o*_(*β*) ≥ 0. Hence,

- If $\underset{\_}{\beta }>{\beta}_2$, we have that for each *β* ∈ [*β*_1_, *β*_2_] it holds that *a*_*o*_(*β*) < 0. According to (10b), this means that the available oxygen in *A* does not allow proliferation and that not all tumor cells in *A* can stay alive. Since *β* is the percentage with which tumor cells rely on glycolysis, in this case, for any proliferation to happen, the available oxygen imposes greater reliance on glycolysis than the maximum, i.e. *β*_2_.
- If $\underset{\_}{\beta}\le {\beta}_2$, we have that for each $\beta \in \left[\max \left(\underset{\_}{\beta },{\beta}_1\right),{\beta}_2\right]$ it holds that *a*_*o*_(*β*) ≥ 0

Investigation of (11): For each *β* ∈ [*β*_1_, *β*_2_] we define the function

$$ {a}_{gl}\left(\beta \right)=\frac{Gl_{av}-{l}_t(A)\frac{17\beta +1}{36}{K}_{ATP}\varDelta \tau}{\left(\lambda -1\right){l}_t(A)\left( cc/\varDelta \tau \right)\frac{17\beta +1}{36}{K}_{ATP}\varDelta \tau} $$

For each given *β* ∈ [*β*_1_, *β*_2_], we observe the following:

11a) If *a*_*gl*_(*β*) ≥ 0: *a*_*gl*_(*β*) is the maximum mitosis rate that the tumor cells can achieve for the specific *β*, subject solely to the limitations imposed by the available glucose in *A*.

11b) If *a*_*gl*_(*β*) < 0, it is implied that ${Gl}_{av}-{l}_t(A)\frac{17\beta +1}{36}{K}_{ATP}\varDelta \tau <0.$ This means that for the specific *β*, no positive mitosis rate can be achieved. In fact, available glucose does not suffice for all tumor cells in *A* to stay alive.

Furthermore,

11c) For each *β* ∈ [0, 1], *a*_*gl*_(*β*) is a decreasing function of *β*.

11d) If a *β* ∈ [*β*_1_, *β*_2_] such that *a*_*gl*_(*β*) ≥ 0 exists, it should also satisfy

$$ \beta \le \frac{1}{17}\left(\frac{36{Gl}_{av}}{l_t(A){K}_{ATP}\varDelta \tau}-1\right)\equiv \overline{\beta} $$

Since *a*_*gl*_(*β*) is decreasing, $\overline{\beta}$ is actually the highest number for which *a*_*gl*_(*β*) ≥ 0. Hence,

- If $\overline{\beta}<{\beta}_1$, then for each *β* ∈ [*β*_1_, *β*_2_] it holds that *a*_*gl*_(*β*) < 0. According to (11b), this means that the available glucose in *A* does not allow proliferation and that not all tumor cells in *A* can stay alive. Since *β* is the percentage with which tumor cells rely on glycolysis, in this case, for any proliferation to happen, the available glucose imposes lower reliance on glycolysis than the minimum, i.e. *β*_1_.
- If $\overline{\beta}\ge {\beta}_1$, we have that for each $\beta \in \left[{\beta}_1,\mathit{\min}\left(\overline{\beta},{\beta}_2\right)\right]$ it holds that *a*_*o*_(*β*) ≥ 0

We are now ready to complete Step 2:

Case 2.2.1. If $\underset{\_}{\beta }>{\beta}_2$ or $\overline{\beta}<{\beta}_1$ or $\min \left(\overline{\beta},{\beta}_2\right)<\max \left(\underset{\_}{\beta },{\beta}_1\right)$, the analysis above implies that for each *β* ∈ [*β*_1_, *β*_2_], the available resources in *A* (oxygen and/or glucose) do not suffice for all tumor cells in *A* to stay alive. We proceed as follows:

We pick a random $\overset{\sim }{\beta }$ ∈[*β*_1_, *β*_2_].

If $\overset{\sim }{\beta}\ne 1$, the number of tumor cells that will remain alive is given by

$$ {N}_c=\mathit{\min}\left(\frac{O_{av}}{\left(\frac{1-\overset{\sim }{\beta }}{6}\right){K}_{ATP}\varDelta \tau},\frac{Gl_{av}}{\left(\frac{17\overset{\sim }{\beta }+1}{36}\right){K}_{ATP}\varDelta \tau}\right) $$

If $\overset{\sim }{\beta }=1$, the number of tumor cells that will remain alive is given by

$$ {N}_c=\frac{Gl_{av}}{\left(\frac{17\overset{\sim }{\beta }+1}{36}\right){K}_{ATP}\varDelta \tau}=\frac{2{Gl}_{av}}{K_{ATP}\varDelta \tau} $$

In any case, the number of tumor cells that will become necrotic is given by

$$ \overline{N_c}={l}_t(A)-{N}_c $$

Hence, for the next time instant we have

$$ {l}_{t+\varDelta \tau}(A)={N}_c $$

$$ {nc}_{t+\varDelta \tau}(A)={nc}_t(A)+\overline{N_c} $$

$$ {o}_{t+\varDelta \tau}(A)={O}_{av}-{N}_c\left(\frac{1-\overset{\sim }{\beta }}{6}\right){K}_{ATP}\varDelta \tau $$

$$ {gl}_{t+\Delta \tau }(A)={Gl}_{av}-{N}_c\left(\frac{17\overset{\sim }{\beta }+1}{36}\right){K}_{ATP}\Delta \tau $$

Case 2.2.2. If $\underset{\_}{\beta}\le {\beta}_2$, $\overline{\beta}\ge {\beta}_1$ and $\min \left(\overline{\beta},{\beta}_2\right)\ge \max \left(\underset{\_}{\beta },{\beta}_1\right)$, i.e. the complement of the condition in Case 2.2.1 holds, according to the preceding analysis we have that for each $\beta \in \left[\max \left(\underset{\_}{\beta },{\beta}_1\right),\min \left(\overline{\beta},{\beta}_2\right)\right]$ there exists a nonnegative mitosis rate *a* such that the inequalities *O*_*c*_ ≤ *O*_*av*_ and *Gl*_*c*_ ≤ *Gl*_*av*_ are satisfied. Again, we pick a random $\overset{\sim }{\beta }$ in $\left[\max \left(\underset{\_}{\beta },{\beta}_1\right),\min \left(\overline{\beta},{\beta}_2\right)\right]$.

If $\overset{\sim }{\beta}\ne 1$, the corresponding mitosis rate is

$$ \overset{\sim }{a}=\mathit{\min}\left(\frac{O_{av}-{l}_t(A)\frac{1-\overset{\sim }{\beta }}{6}{K}_{ATP}\varDelta \tau}{\left(\lambda -1\right){l}_t(A)\left( cc/\varDelta \tau \right)\frac{1-\overset{\sim }{\beta }}{6}{K}_{ATP}\varDelta \tau},\frac{Gl_{av}-{l}_t(A)\frac{17\overset{\sim }{\beta }+1}{36}{K}_{ATP}\varDelta \tau}{\left(\lambda -1\right){l}_t(A)\left( cc/\varDelta \tau \right)\frac{17\overset{\sim }{\beta }+1}{36}{K}_{ATP}\varDelta \tau},{a}_{max}\right) $$

If $\overset{\sim }{\beta }=1$, the corresponding mitosis rate is

$$ \overset{\sim }{a}=\mathit{\min}\left(\frac{Gl_{av}-{l}_t(A)\frac{17\overset{\sim }{\beta }+1}{36}{K}_{ATP}\varDelta \tau}{\left(\lambda -1\right){l}_t(A)\left( cc/\varDelta \tau \right)\frac{17\overset{\sim }{\beta }+1}{36}{K}_{ATP}\varDelta \tau},{a}_{max}\right) $$

$$ =\mathit{\min}\left(\frac{Gl_{av}-\frac{1}{2}{l}_t(A){K}_{ATP}\varDelta \tau}{\frac{1}{2}\left(\lambda -1\right){l}_t(A)\left( cc/\varDelta \tau \right){K}_{ATP}\varDelta \tau},{a}_{max}\right) $$

Hence, for the next time instant we have

$$ {l}_{t+\varDelta \tau}(A)={l}_t(A)+\overset{\sim }{a}{l}_t(A) $$

$$ {nc}_{t+\varDelta \tau}(A)={nc}_t(A) $$

$$ {o}_{t+\varDelta \tau}(A)={O}_{av}-{l}_t(A)\frac{1-\overset{\sim }{\beta }}{6}{K}_{ATP}\varDelta \tau -\left(\lambda -1\right)\overset{\sim }{a}{l}_t(A)\left( cc/\varDelta \tau \right)\frac{1-\overset{\sim }{\beta }}{6}{K}_{ATP}\varDelta \tau $$

$$ {gl}_{t+\Delta \tau }(A)={Gl}_{av}-{l}_t(A)\frac{17\overset{\sim }{\beta }+1}{36}{\mathrm{K}}_{ATP}\Delta \tau -\left(\lambda -1\right)\overset{\sim }{a}{l}_t(A)\left( cc/\Delta \tau \right)\frac{17\overset{\sim }{\beta }+1}{36}{\mathrm{K}}_{ATP}\Delta \tau $$

This concludes our analysis. Το summarize, tumor cells may be able to rely on glycolysis within certain limits, i.e. *β*_1_ and *β*_2_, but these limits may become narrower by the available quantities of glucose and oxygen; in that case, the ability of tumor cells to proliferate with high mitosis rates is impaired. Mathematically, this is reflected by the decreased probability to attain high mitosis rates, or even the inability of tumor cells to stay alive.

We note that the calculation of *O*_*av*_ and *Gl*_*av*_ in Step 1 implies that normal cells are the first to fulfill their needs by the existing resources. This is certainly not accurate; again, a more realistic approach would be to introduce some randomness in the percentage of resources that would be available for normal versus tumor cells. Α random number roughly proportional to the ratio normal/tumor cells in the voxel may be a reasonable choice.

### VI. The effects of tumor-induced vascular remodeling

In this section, we will propose a method to quantify the effects of tumor-induced vascular remodeling, in terms of how it affects the local provision of oxygen and glucose by the vascular system. The quantification we propose is based on the basic physiology of the vascular network plus additional biological observations regarding how it is affected by tumor growth.

Ideally, the vascular network works like a buffer of nutrients, in our case, oxygen and glucose. If, at a specific time instant, the concentration of a substance dissolved in the blood is higher than the respective concentration in the surrounding tissue, the substance diffuses through the vessel walls towards the tissue until the two concentrations are equal, and vice versa. The speed of this diffusion process as well as the capability of the local vascular network to quickly balance these concentrations is limited by the vessel density and total surface of the blood vessel walls in the region under consideration [92].

In our model, we will assume that the concentrations of dissolved oxygen and glucose in the blood are constant. In a voxel *A* of specific volume, these concentrations correspond to quantities of oxygen and glucose within *A*, which we denote by $\overline{o_0}$ and $\overline{gl_0}$. If at a specific time *t*, the quantity of say, oxygen in *A* i.e. *o*_*t*_(*A*) is lower (higher) than $\overline{o_0}$, this implies that the concentration of oxygen in *A* is lower (higher) than the concentration of dissolved oxygen in the blood. Hence, the provision of oxygen in *A* i.e. *o*_*b*_*t*_(*A*) should increase (decrease) to level this imbalance. We model this increase (decrease) during each time step by a random fraction of the quantity $\left(\left(\ \overline{o_0}-{o}_t(A)\ \right)/\varDelta \tau \right)$. The respective quantity $\left(\left(\ \overline{gl_0}-{gl}_t(A)\ \right)/\varDelta \tau \right)$ is used for glucose. This results in the following equations:

$$ o\_{b}_{t+\varDelta \tau}(A)=o\_{b}_t(A)+{r}_1\left(\left(\ \overline{o_0}-{o}_t(A)\ \right)/\varDelta \tau \right) $$

$$ gl\_{b}_{t+\varDelta \tau}(A)= gl\_{b}_t(A)+{r}_2\left(\left(\ \overline{gl_0}-{gl}_t(A)\ \right)/\varDelta \tau \right) $$

where *o*_*b*_*t* + *Δτ*_(*A*), *gl*_*b*_*t* + *Δτ*_(*A*) are the oxygen and glucose supply rates (pmols/sec) by the local vascular network within *A* during the time interval *t* → *t* + *Δτ*. The quantities *o*_*b*_*t*_(*A*) and *gl*_*b*_*t*_(*A*) are the respective supply rates during the previous time interval, i.e. *t* − *Δτ* → *t*. The numbers *r*_1_ and *r*_2_ are random numbers uniformly distributed in the interval [0, 1].

We note that *o*_*b*_*t*_(*A*) and *gl*_*b*_*t*_(*A*) may take negative values. This simply reflects the fact that when the respective substance concentration in *A* is higher than the one in the blood and the consumption of the substance within *A* is low enough, diffusion may happen towards the vessels, decreasing thereby the substance quantity in the surrounding tissue.

It is clear, however, that *o*_*b*_*t*_(*A*) and *gl*_*b*_*t*_(*A*) cannot grow unboundedly neither towards positive nor towards negative values. As previously mentioned, they are limited by the local vessel density and total surface of the blood vessel walls in *A*. A detailed quantitative analysis addressing the pertinent mechanisms would render the model extremely complex. We therefore opt for a more macroscopic approach.

In [93] a maximum value of oxygen consumption for normal mammalian cells is given. From this, an upper bound for the absolute value of *o*_*b*_*t*_(*A*) can be deduced. Assuming that in the tissue under consideration, normal cell metabolism does not change and utilizes oxygen and glucose in a steady ratio, we can deduce a similar bound for the absolute value of *gl*_*b*_*t*_(*A*). We denote these two constant numbers by *o*_*b*_*max* and *gl*_*b*_*max*. In normal tissue, these numbers remain constant and are the same for each voxel. In our case, however, these bounds may be different for each voxel and are subject to a temporal evolution, inflicted by the tumor-induced vessel regression and angiogenesis. To address this in our model, we introduce the *N*^3^ × 1 vectors *o*_*b*_ *max*_*t*_ and *gl*_*b*_ *max*_*t*_. Consistent with our previous notation, *o*_*b*_*max*_*t*_(*A*) and *gl*_*b*_*max*_*t*_(*A*) denote the aforementioned bounds for the voxel *A* during the time interval *t* − *Δτ* → *t*. For each voxel *A*, the initial values of *o*_*b*_*max*_*t*_(*A*) and *gl*_*b*_*max*_*t*_(*A*) at time *t* = 0 are respectively the constants *o*_*b*_*max* and *gl*_*b*_*max*. Essentially, *o*_*b*_*max*_*t*_(*A*) and *gl*_*b*_*max*_*t*_(*A*) quantify the capacity of the vascular network within *A* to provide/absorb molecules to/from the surrounding tissue, leveling thereby the concentration imbalances between blood and tissue. A disorganized and regressed vascular network in *A* implies lower values for *o*_*b*_*max*_*t*_(*A*) and *gl*_*b*_*max*_*t*_(*A*), as is usually the case in the interior of a tumor. A robust, dense vascular network in *A* implies higher values for *o*_*b*_*max*_*t*_(*A*) and *gl*_*b*_*max*_*t*_(*A*), as is the case for the outer proliferating rim of a tumor.

In what follows, we will use the temporal evolution of these quantities in each voxel to quantify the effects of tumor-induced vascular remodeling on the local provision/absorption of nutrients. We start from some basic biological background.

It is well documented, that as tumors grow, they co-opt and affect the pre-existing host vasculature by a number of ways, for which the collective term tumor-induced vascular remodeling is commonly used. Tumor-induced vascular remodeling consists in several mechanisms, including vessel occlusion, disintegration, and new vessel creation. The latter is most commonly referred as tumor-induced angiogenesis, and results in a tortuous, highly irregular vascular network [4, 6, 7].

In [94, 95] the authors observed that cancer cells initially co-opt host existing vasculature and grow as well vascularized tumors for several days, up to 2 mm in diameter. No evidence of angiogenesis is observed during this period. Progressively, blood vessels near the tumor core start to regress and/or become occluded, while tumor periphery displays robust angiogenesis. Later work in [96, 97] demonstrated that this pattern repeats itself during later stages of tumor growth; once the tumor grows over a well vascularized region, local vasculature starts to regress. At the same time, tumor periphery displays high angiogenic activity, thereby further promoting tumor growth.

The exact biological mechanisms pertaining to these phenomena are not well understood. Generally, they are attributed to a variety of complex molecular and biomechanical interactions between existing vasculature and tumor cells. It is evident, however, that tumor-induced vascular remodeling affects the local supply of nutrients in tumors and this is where we are going to focus. Taking into consideration the aforementioned evidence, we will try to quantify the spatiotemporal evolution of local oxygen and glucose provision, i.e. the vectors *o*_*b*_*t*_ , *gl*_*b*_*t*_ , *o*_*b*_*max*_*t*_ and *gl*_*b*_*max*_*t*_

Since tumor-induced vascular remodeling occurs either in the interior or in the close vicinity of a tumor, at each time step we consider only the voxels that have already been reached by the tumor, that is, voxels for which *l*_*t*_(*A*) + *nc*_*t*_(*A*) > 0. Let *A* denote such a voxel.

According to the aforementioned evidence, vessel regression should decrease *o*_*b*_*max*_*t*_(*A*) and *gl*_*b*_*max*_*t*_(*A*). As the occupation of *A* by tumor cells (live or necrotic) increases, the vessel regression rate should also increase, and hence, *o*_*b*_*max*_*t*_(*A*) and *gl*_*b*_*max*_*t*_(*A*) should decrease at a higher rate.

On the other hand, angiogenesis should increase *o*_*b*_*max*_*t*_(*A*) and *gl*_*b*_*max*_*t*_(*A*). A lower occupation of *A* by live tumor, necrotic tumor and necrotic normal cells, implies a higher angiogenesis rate and hence, a higher rate by which *o*_*b*_*max*_*t*_(*A*) and *gl*_*b*_*max*_*t*_(*A*) increase.

Furthermore, tumor angiogenesis is most commonly associated with nutrient deficit, i.e. when oxygen or glucose quantities fall below certain thresholds known respectively as hypoxia and hypoglycemia thresholds. Typical values for these thresholds are 0.30* *o*_0_ and 0.50* *gl*_0_ [5, 37, 98]. We introduce an additional *N*^3^ × 1 logical vector, denoted by *sw*_*t*_ with the following use: at the end of each time step, for each voxel *A*, the quantities *o*_*t*_(*A*) and *gl*_*t*_(*A*) are compared with their respective thresholds; if either of them is below its threshold and the voxel contains live tumor cells, *sw*_*t*_(*A*) is set to 1, indicating that angiogenesis is on for this voxel. Otherwise, *sw*_*t*_(*A*) is set to 0.

Let *v*_*r*_ and *v*_*e*_ denote the maximum rates by which the capacity of the vascular network in *A* to provide/absorb molecules to/from the surrounding tissue (as modeled by *o*_*b*_*max*_*t*_(*A*) and *gl*_*b*_*max*_*t*_(*A*)) decreases or increases, respectively. The orders of magnitude of the corresponding half- and doubling times can be deduced from [94, 95] and are in the order of days.

According to the aforementioned biological evidence and assumptions, a general way to quantify the timely evolution of the macroscopic variables under consideration is

$$ {\displaystyle \begin{array}{c}\begin{array}{l}o\_b\_{\max}_{t+\Delta \tau }(A)=\\ {}\left(1-{f}_r\left({l}_t(A),{nc}_t(A)\right){v}_r+{sw}_t(A)\ {f}_e\left({l}_t(A),{nc}_t(A),{nn}_t(A)\right){v}_e\right).o\_b\_{\max}_t(A)\end{array}\\ {}\begin{array}{l} gl\_b\_{\max}_{t+\Delta \tau }(A)=\\ {}\left(1-{f}_r\left({l}_t(A),{nc}_t(A)\right){v}_r+{sw}_t(A){f}_e\left({l}_t(A),{nc}_t(A),{nn}_t(A)\right){v}_e\right). gl\_b\_{\max}_t(A)\end{array}\end{array}} $$

where *f*_*r*_(*l*_*t*_(*A*), *nc*_*t*_(*A*)) and *f*_*e*_(*l*_*t*_(*A*), *nc*_*t*_(*A*), *nn*_*t*_(*A*)) are functions taking values in [0, 1]. Function *f*_*r*_(*l*_*t*_(*A*), *nc*_*t*_(*A*)) is increasing in both of its arguments. Function *f*_*e*_(*l*_*t*_(*A*), *nc*_*t*_(*A*), *nn*_*t*_(*A*)) is decreasing in all three of its arguments.

These deterministic equations constitute only a rough approximation of the involved dynamics. For a more robust approach, we introduce randomness in them in the following way. Since *v*_*r*_ and *v*_*e*_ are the maximum rates of vessel regression/expansion, at each time step, and for each voxel *A* which has been reached by the tumor, we pick two random numbers *r*_3_ and *r*_4_ in the interval [0, 1] and introduce the more general, stochastic equations

$$ {\displaystyle \begin{array}{c}\begin{array}{l}o\_b\_{\max}_{t+\Delta \tau }(A)=\\ {}\left(1-{f}_r\left({l}_t(A),{nc}_t(A)\right){r}_3{v}_r+{sw}_t(A)\kern0.5em {f}_e\left({l}_t(A),{nc}_t(A),{nn}_t(A)\right){r}_4{v}_e\right)\\ {}.o\_b\_{\max}_t(A)\end{array}\\ {}\begin{array}{l} gl\_b\_{\max}_{t+\Delta \tau }(A)=\\ {}\left(1-{f}_r\left({l}_t(A),{nc}_t(A)\right){r}_3{v}_r+{sw}_t(A){f}_e\left({l}_t(A),{nc}_t(A),{nn}_t(A)\right){r}_4{v}_e\right)\\ {}. gl\_b\_{\max}_t(A)\end{array}\end{array}} $$

It remains to choose the functions *f*_*r*_ and *f*_*e*_. Since the monotonicity of these functions is determined, it remains to choose their shape, i.e. linear, convex or concave. For the simulations presented below, we have chosen functions of linear shape. Putting everything together, to calculate *o*_*b*_*max*_*t* + *Δτ*_(*A*) and *gl*_*b*_*max*_*t* + *Δτ*_(*A*) we will use the equations

$$ {\displaystyle \begin{array}{c}\begin{array}{l}o\_b\_{\max}_{t+\Delta \tau }(A)=\\ {}\left(1-\frac{l_t(A)+{nc}_t(A)}{M}{r}_3{v}_r+{sw}_t(A)\kern0.5em \frac{M-{l}_t(A)-{nc}_t(A)-{nn}_t(A)}{M}{r}_4{v}_e\right)\cdot \\ {}o\_b\_{\max}_t(A)\end{array}\\ {}\begin{array}{l} gl\_b\_{\max}_{t+\Delta \tau }(A)=\\ {}\left(1-\frac{l_t(A)+{nc}_t(A)}{M}{r}_3{v}_r+{sw}_t(A)\frac{M-{l}_t(A)-{nc}_t(A)-{nn}_t(A)}{M}{r}_4{v}_e\right)\cdot \\ {} gl\_b\_{\max}_t(A)\end{array}\end{array}} $$

We note that algorithmically, before each such calculation, a sanity check should be performed for the each of the quantities $\frac{l_t(A)+{nc}_t(A)}{M}$ and $\frac{M-{l}_t(A)-{nc}_t(A)-{nn}_t(A)}{M}$ ensuring that their values stay respectively below 1 and above 0.

To summarize, to model the effects of tumor-induced vascular remodeling, we introduced two additional vectors, that is, two additional variables for each voxel *A*, namely *o*_*b*_*max*_*t*_(*A*) and *gl*_*b*_*max*_*t*_(*A*). These variables quantify the maximum values the local provision/absorption of oxygen and glucose may attain, i.e. the maximum absolute values of *o*_*b*_*t*_(*A*) and *gl*_*b*_*t*_(*A*) reflecting thereby the capacity of the local vascular network to provide/absorb molecules to/from the surrounding tissue. The spatiotemporal evolution of these variables reflects the effects of vessel regression and angiogenesis induced by the tumor. The resulting algorithm applied at each time step for each voxel *A* follows:

Note: For any positive number *μ* and *xϵ ℝ* we will make use of the function

$$ {B}_{\mu }(x)=\left\{\begin{array}{c}-\mu, x<-\mu \\ {}\ x,-\mu \le x\le \mu\ \\ {}\mu, x>\mu \end{array}\right. $$

Case 1: If *l*_*t*_(*A*) = *nc*_*t*_(*A*) = 0 that is, the tumor has not yet reached *A*. In this case, the maximum absolute values of oxygen and glucose provision/absorption during the time step *t* → *t* + *Δτ* simply equal the ones during the previous time step, *t* − *Δτ* → *t*:

$$ o\_b\_{\mathit{\max}}_{t+\varDelta \tau}(A)=o\_b\_{\mathit{\max}}_t(A) $$

$$ gl\_b\_{\mathit{\max}}_{t+\varDelta \tau}(A)= gl\_b\_{\mathit{\max}}_t(A) $$

We pick two random numbers *r*_1_, *r*_2_, uniformly distributed in [0, 1]. The actual provision/absorption for oxygen and glucose by the vascular system during time interval *t* → *t* + *Δτ* is calculated by

$$ o\_{b}_{t+\varDelta \tau}(A)={B}_{o\_b\_{\mathit{\max}}_{t+\varDelta \tau}(A)}\left(\ o\_{b}_t(A)+{r}_1\left(\left(\ \overline{o_0}-{o}_t(A)\ \right)/\varDelta \tau \right)\ \right) $$

$$ gl\_{b}_{t+\varDelta \tau}(A)={B}_{\mathrm{gl}\_b\_{\mathit{\max}}_{t+\varDelta \tau}(A)}\left(\  gl\_{b}_t(A)+{r}_2\left(\left(\ \overline{gl_0}-{gl}_t(A)\ \right)/\varDelta \tau \right)\ \right) $$

Case 2: If *l*_*t*_(*A*) + *nc*_*t*_(*A*) > 0 that is, the tumor has reached *A*. We first pick two random numbers *r*_1_, *r*_2_, uniformly distributed in [0, 1] and calculate

$$ \overline{o\_{b}_{t+\varDelta \tau}(A)}=o\_{b}_t(A)+{r}_1\left(\left(\ \overline{o_0}-{o}_t(A)\ \right)/\varDelta \tau \right) $$

$$ \overline{gl\_{b}_{t+\varDelta \tau}(A)}= gl\_{b}_t(A)+{r}_2\left(\left(\ \overline{gl_0}-{gl}_t(A)\ \right)/\varDelta \tau \right) $$

Again, we pick two random numbers *r*_3_, *r*_4_ uniformly distributed in [0, 1]. The upper bounds for the absolute values of *o*_*b*_*t* + *Δτ*_(*A*) and *gl*_*b*_*t* + *Δτ*_(*A*) are given by

$$ {\displaystyle \begin{array}{c}\begin{array}{l}o\_b\_{\max}_{t+\Delta \tau }(A)=\\ {}\left(1-\frac{l_t(A)+{nc}_t(A)}{M}{r}_3{v}_r+{sw}_t(A)\kern0.5em \frac{M-{l}_t(A)-{nc}_t(A)-{nn}_t(A)}{M}{r}_4{v}_e\right)\cdot o\_b\_{\max}_t(A)\end{array}\\ {}\begin{array}{l} gl\_b\_{\max}_{t+\Delta \tau }(A)=\\ {}\left(1-\frac{l_t(A)+{nc}_t(A)}{M}{r}_3{v}_r+{sw}_t(A)\frac{M-{l}_t(A)-{nc}_t(A)-{nn}_t(A)}{M}{r}_4{v}_e\right)\cdot gl\_b\_{\max}_t(A)\end{array}\end{array}} $$

The provision/absorption for oxygen and glucose by the vascular system during time interval *t* → *t* + *Δτ* is calculated by

$$ o\_{b}_{t+\varDelta \tau}(A)={B}_{o\_b\_{\mathit{\max}}_{t+\varDelta \tau}(A)}\left(\overline{\ o\_{b}_{t+\varDelta \tau}(A)}\ \right) $$

$$ gl\_{b}_{t+\varDelta \tau}(A)={B}_{gl\_b\_{\mathit{\max}}_{t+\varDelta \tau}(A)}\left(\overline{\  gl\_{b}_{t+\varDelta \tau}(A)}\ \right) $$

### VII. The complete model architecture. Modularity and adjustability

The model we propose can be seen as a discrete time dynamical system. The state of the system consists of the nine *N*^3^ × 1 vectors *l*_*t*_, *nc*_*t*_, *nn*_*t*_, *o*_*t*_, *gl*_*t*_, *o*_*b*_*t*_, *gl*_*b*_*t*_, *o*_*b*_*max*_*t*_ and *gl*_*b*_*max*_*t*_

In sections I-VI we have defined the following operators.

- The operator defined in section VI, which we denote by *F*_*vr*_. Applying this operator to the state vector consists in applying the algorithm described in the last section for each voxel. This operator calculates the supply rate of oxygen and glucose during the time interval *t* → *t* + *Δτ* from the respective values during the previous time interval, *t* − *Δτ* → *t*, as it is dictated by the effects of tumor-induced vascular remodeling.
- The operator defined algorithmically in section V, which we denote by *F*_*pn*_. Applying operator *F*_*pn*_ to the state vector, consists in checking all cases described in section V and performing the respective calculations for each voxel in the lattice. This operator calculates the proliferation/necrosis of cells in each voxel, as they are dictated by the voxels’ oxygen and glucose levels and supply rates.
- The operators defined in section III, which we denote by *F*_*o*_, *F*_*gl*_ . Applying each of these operators to the state vector consists in multiplying the matrices (*T*_*o*_ or *T*_*gl*_) with the respective vector *o*_*t*_ or *gl*_*t*_, thereby calculating how chemical fields change due to diffusion.
- The operator defined algorithmically in section IV, which we denote by *F*_*c*_. Applying the operator *F*_*c*_ to the state vector consists in executing Algorithm 2, calculating thereby how cancer cell populations within each voxel change due to cell diffusion.

**Fig. 2:**
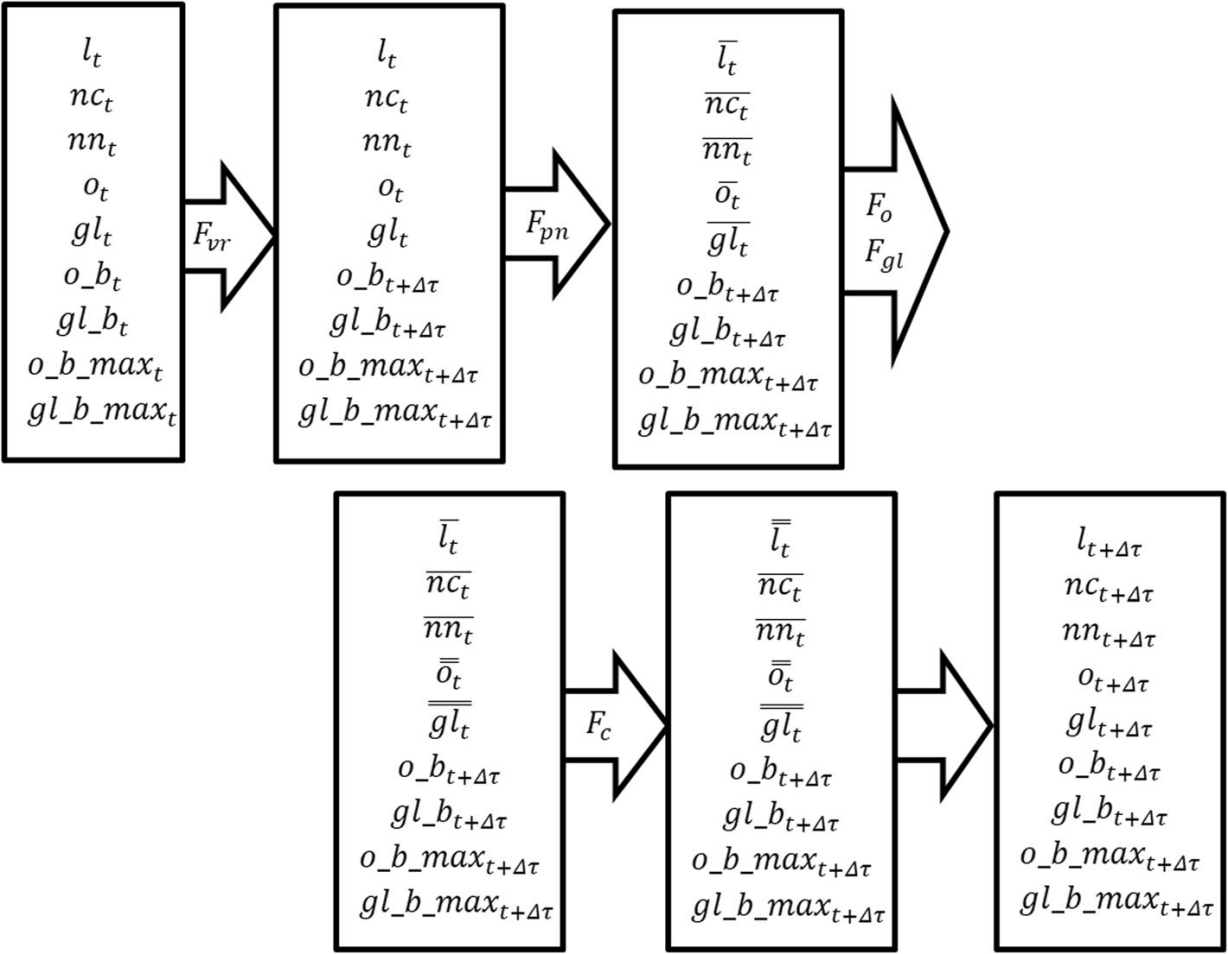
The complete model architecture

Knowledge of the state vector at time *t* allows us to calculate the state vector at time *t* + *Δτ*, by applying the algorithm depicted in Fig. 2.

Phenomena pertaining to tumor-induced vascular remodeling, nutrient consumption, cell proliferation and cellular or molecular diffusion are modeled by separate operators (i.e. algorithmic modules), applied sequentially to the state vector. Within the proposed methodology, the algorithmic modules corresponding to operators *F*_*pn*_, *F*_*vr*_ are completely re-adjustable. This facilitates the simulation of scenarios based on different hypotheses concerning the effects of tumor-induced vascular remodeling on nutrient supply rates, cell proliferation, necrosis and metabolism of chemical species. A variety of choices is also available for re-adjusting *F*_*c*_; we provide some suggestions in the appendix. Introduction of additional diffusion operators like *F*_*o*_ and *F*_*gl*_ and extension of operators *F*_*pn*_ and *F*_*vr*_ enables the consideration of additional chemical species such as lactate, growth factors and chemotherapeutic agents. Gradual removal of necrotic cells from the tumor mass may (and should) also be considered. Introduction of additional cellular species is also feasible, by considering additional cellular diffusion operators and appropriate readjustment of *F*_*pn*_.

Of note, there is a large disparity between the time scales of chemical and cellular diffusion, with the latter evolving much more slowly. The diffusion coefficients of oxygen and glucose are in the order of 10^−5^ cm^2^/sec while the respective coefficient for tumor cells is in the order of 10^−8^ cm^2^/sec. Furthermore, tumor cell diffusion in neighboring voxels is also affected by their proliferation. This allows applying *F*_*c*_ to the state vector every several time steps *κ*; for the simulations presented in the next section, we used a time step *Δτ*= 10 s and *κ*= 30 (i.e. 5 mins).

The methodology we described in the previous sections essentially casts the problem of modeling tumor growth in the realm of spatially distributed, stochastic dynamical systems. The state of the system evolves according to a law of the form *x*_*k* + 1_ = *f*(*x*_*k*_) where the transition *x*_*k*_ → *x*_*k* + 1_ is stochastic and *x* has an additional spatial structure.

The model was implemented by making extensive use of MATLAB’s vectorized approach to coding. We note that apart from the operators *F*_*o*_, *F*_*gl*_ and *F*_*c*_, this vectorized implementation is feasible also for the operators *F*_*vr*_ and *F*_*pn*_. However, to keep the code readable, in this work we have opted to implement *F*_*vr*_ and *F*_*pn*_ using loops. Although in the present work we did not exploit multicore computation, it is clear that each of the aforementioned algorithmic operators is eligible for parallel implementation. Furthermore, vectorized implementation of the resulting algorithmic modules opens the road for exploiting the capabilities of modern tools like Python’s Numba compiler or TensorFlow for computation on GPUs. This will facilitate simulations over larger tissue areas with finer spatial and temporal resolutions, plus, importantly, a comparative analysis of the numerical error induced by the discretization parameters. We note that such an analysis has not been performed yet, since it requires the consideration of more and smaller values for *Δs* and *Δt*. This, however, increases significantly the computational burden of each simulation, and requires a completely different implementation of the model in terms of programming. It is therefore left for future work.

### VIII. Simulations and use cases

In this section, we use the model developed in the previous sections for a theoretical study of tumor growth, consisting of two parts. First, we use the model to visualize various aspects of tumor expansion. The resulting images are qualitatively compared with pertinent biological observations. We then perform a multivariate analysis regarding the effects of a subset of model parameters on the number of live cancer cells after a certain period of free growth.

**Fig. 3:**
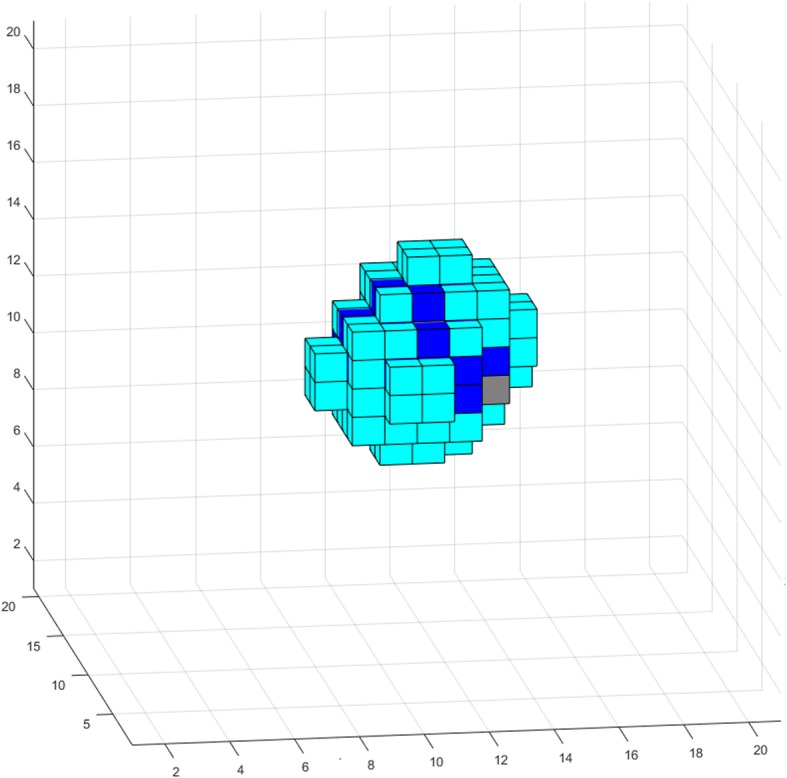
Visualization of tumor growth at the 70th day

**Fig. 4:**
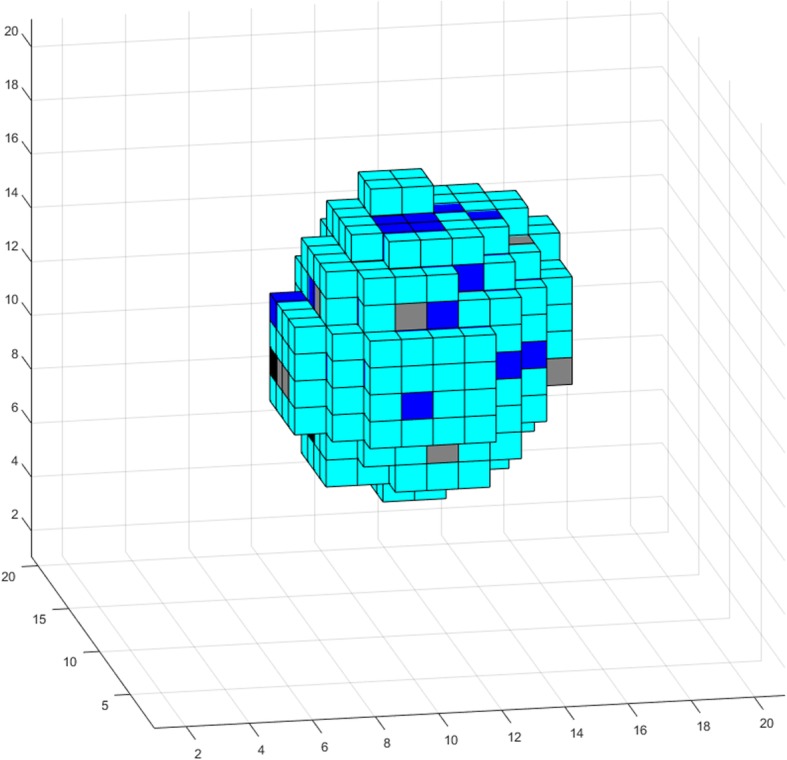
Visualization of tumor growth at the 90th day

**Fig. 5:**
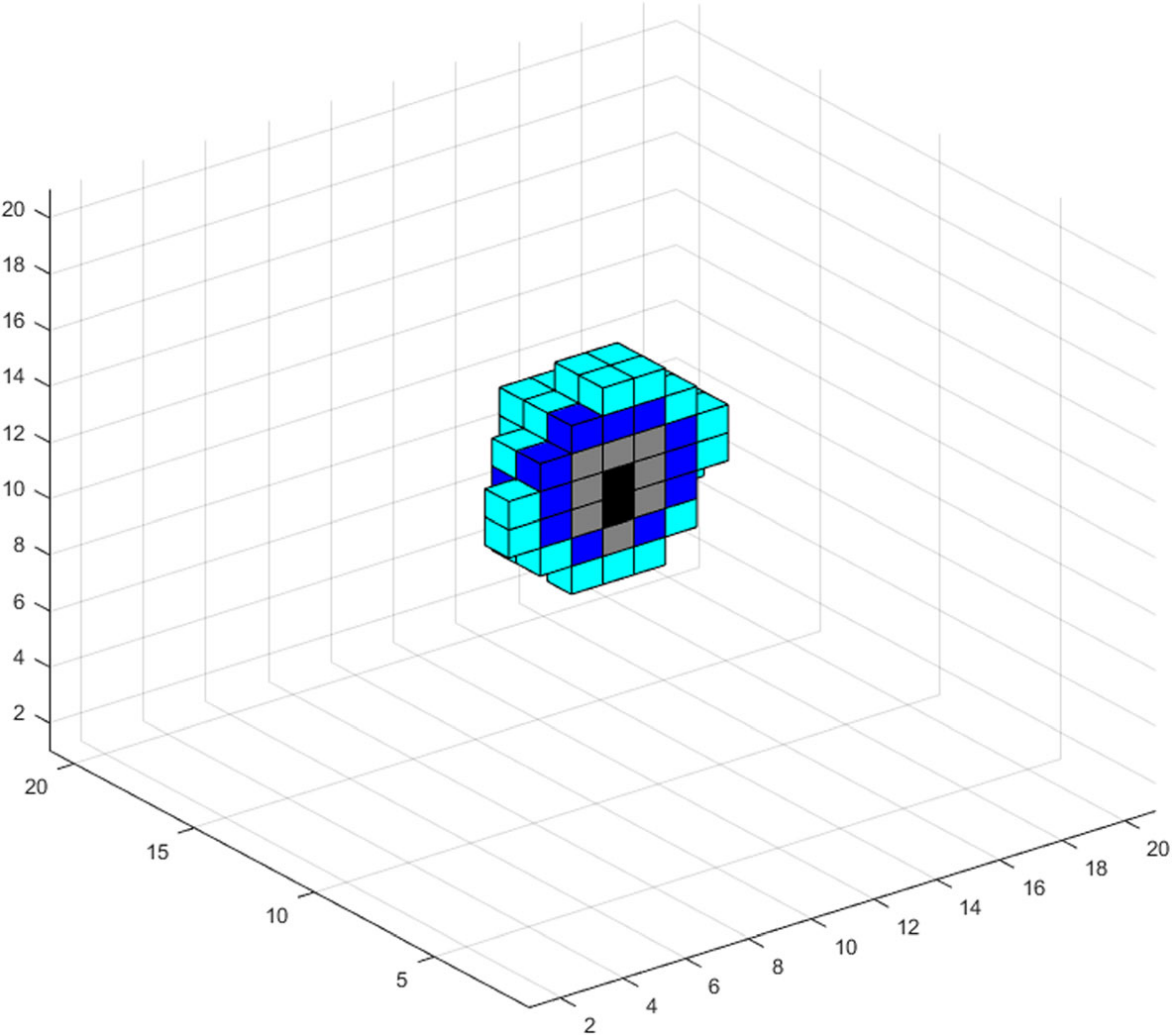
Visualization of tumor growth at the 70th day, vertical section

**Fig. 6:**
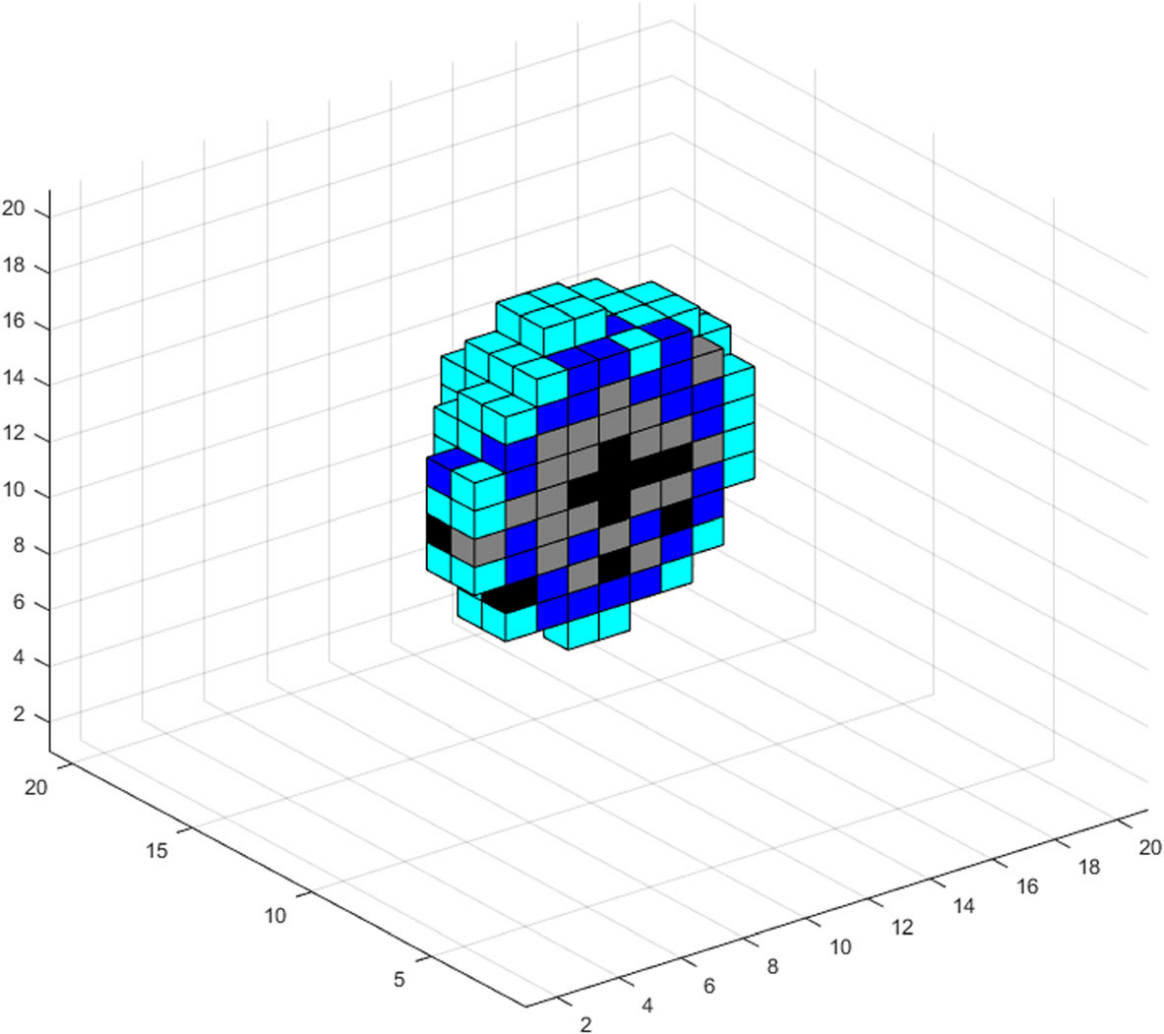
Visualization of tumor growth at the 90th day, vertical section

**Fig. 7:**
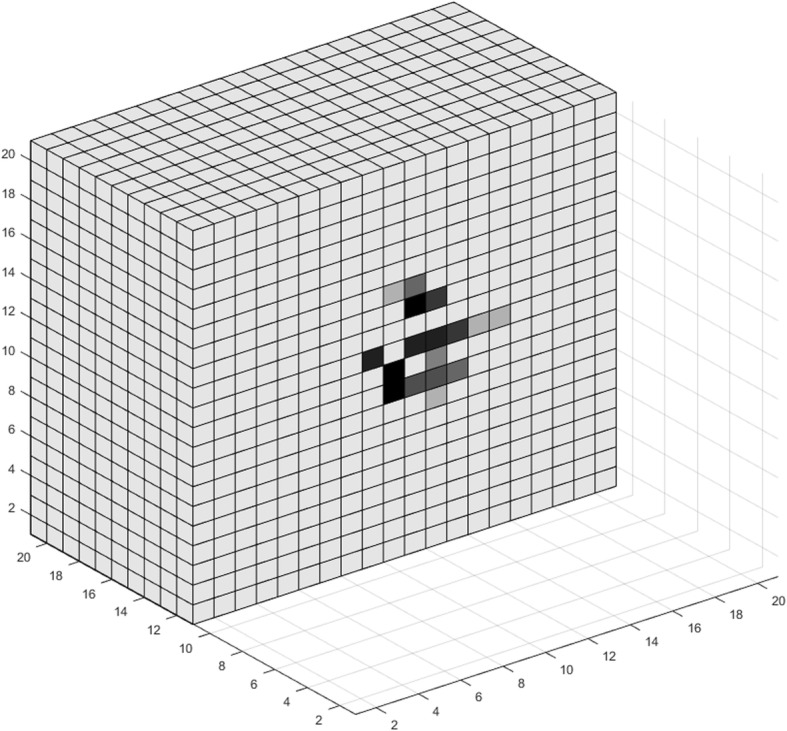
Oxygen levels per voxel at the 70th day. Darker color implies lower oxygen

**Fig. 8:**
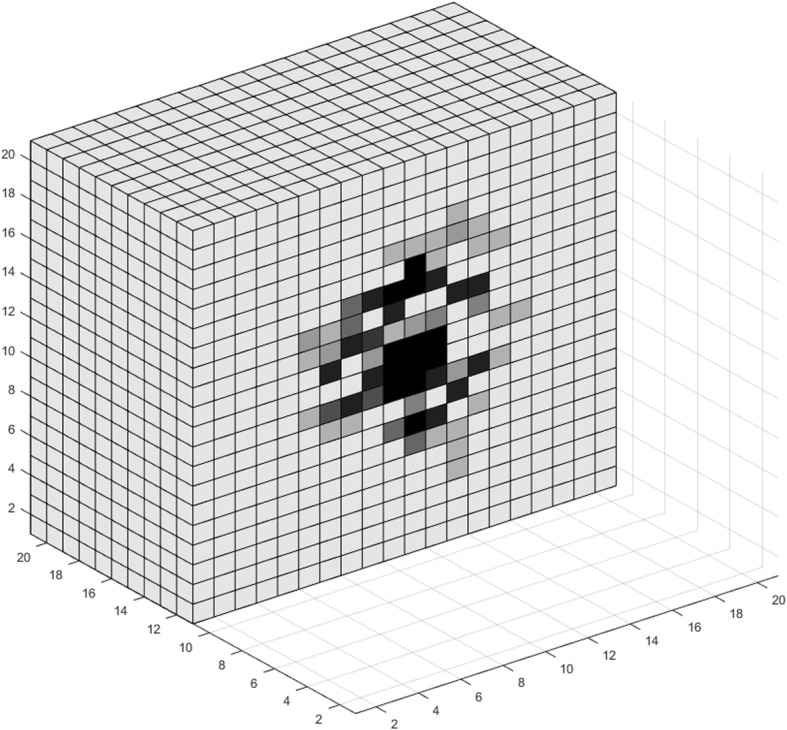
Oxygen levels per voxel at the 90th day. Darker color implies lower oxygen

**Fig. 9:**
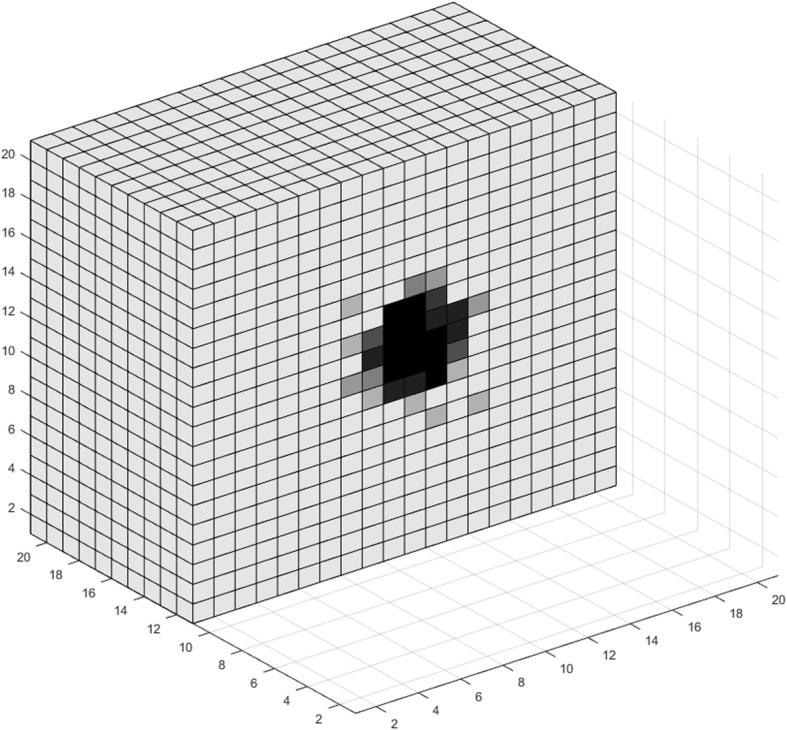
Glucose levels per voxel at the 70th day. Darker color implies lower glucose

**Fig. 10:**
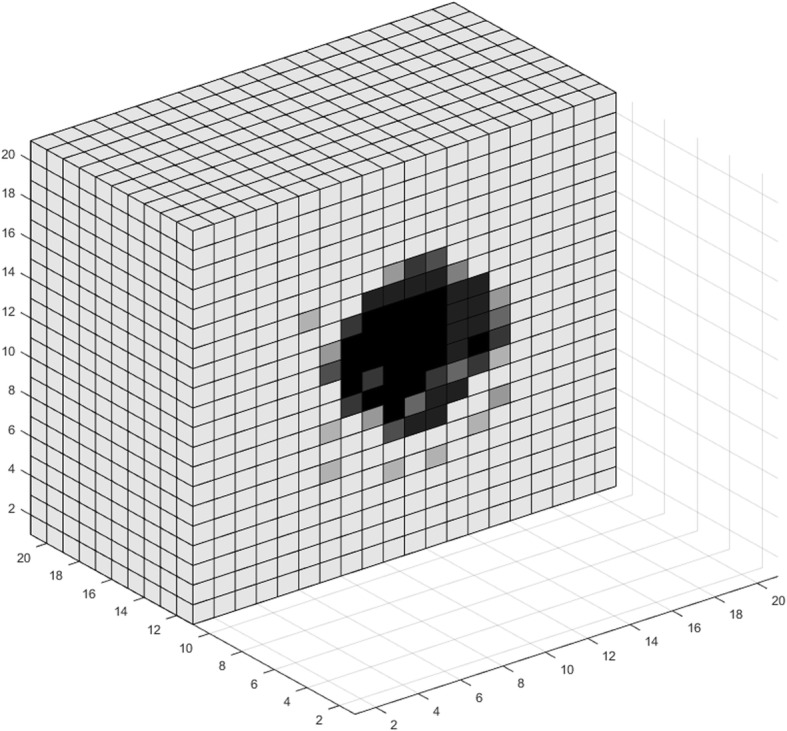
Glucose levels per voxel at the 90th day. Darker color implies lower glucose

**Fig. 11:**
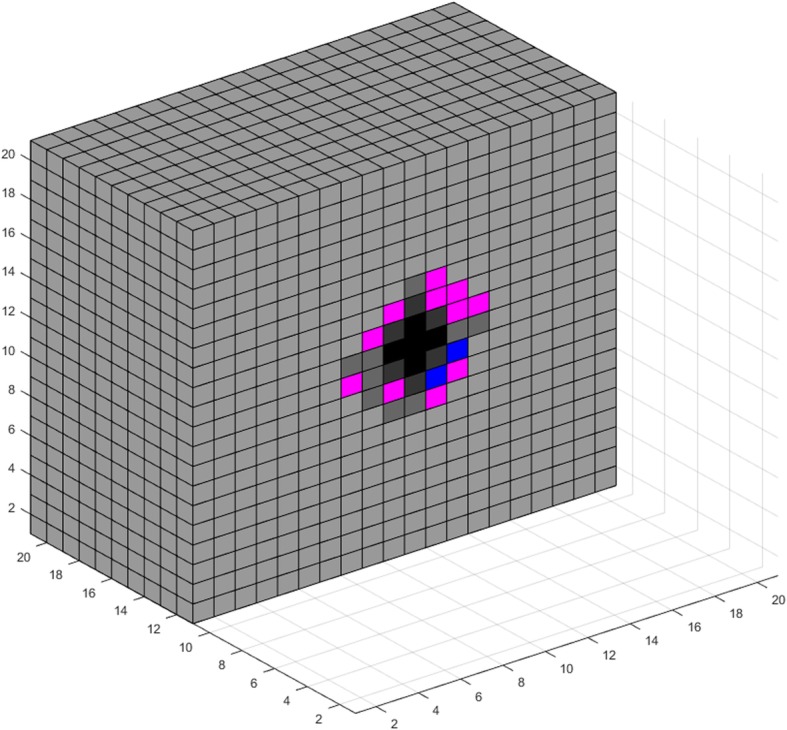
Capacity of the vascular network per voxel to provide/absorb nutrients to/from the surrounding tissue, 70th day

**Fig. 12:**
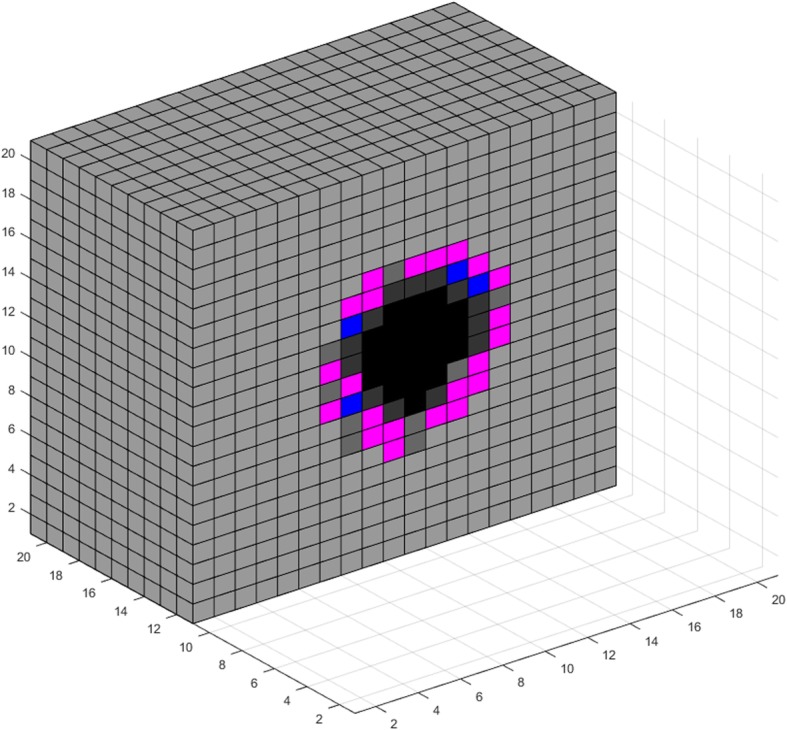
Capacity of the vascular network per voxel to provide/absorb nutrients to/from the surrounding tissue, 90th day

The proposed model can be used for visualizing various phenomena encountered during the expansion of a tumor. Figures 3, 4, 5, 6, 7, 8, 9, 10, 11 and 12, which we explain below, depict a series of such examples. These images are snapshots of a simulation with the following parameter values (see also sections about metabolism and tumor-induced vascular remodeling above). Maximum mitosis rate for cancer cells *a*_*max*_ was set such that their minimum doubling time is 5 days. Parameter lambda is set *λ* = 10.

Maximum vasculature regression rate (*v*_*r*_) was set such that the corresponding minimum halftime time is 5 days. Maximum vasculature expansion rate (*v*_*e*_) was set such that the corresponding minimum doubling time is 1 day. A spatially constant, synthetic diffusion tensor was used for the diffusion of live cancer cells, defined by the orthonormal vectors

$$ \left\{{\left(\raisebox{1ex}{$\mathbf{1}$}\!\left/ \!\raisebox{-1ex}{$\mathbf{2}$}\right.\ \raisebox{1ex}{$\mathbf{1}$}\!\left/ \!\raisebox{-1ex}{$\mathbf{2}$}\right.\ \raisebox{1ex}{$\sqrt{\mathbf{2}}$}\!\left/ \!\raisebox{-1ex}{$\mathbf{2}$}\right.\right)}^{\boldsymbol{T}},{\left(-\raisebox{1ex}{$\mathbf{1}$}\!\left/ \!\raisebox{-1ex}{$\mathbf{2}$}\right.-\raisebox{1ex}{$\mathbf{1}$}\!\left/ \!\raisebox{-1ex}{$\mathbf{2}$}\right.\ \raisebox{1ex}{$\sqrt{\mathbf{2}}$}\!\left/ \!\raisebox{-1ex}{$\mathbf{2}$}\right.\right)}^{\boldsymbol{T}},{\left(\raisebox{1ex}{$\sqrt{\mathbf{2}}$}\!\left/ \!\raisebox{-1ex}{$\mathbf{2}$}\right.-\raisebox{1ex}{$\sqrt{\mathbf{2}}$}\!\left/ \!\raisebox{-1ex}{$\mathbf{2}$}\right.\ \mathbf{0}\right)}^{\boldsymbol{T}}\right\}. $$

The corresponding quantities $\sqrt{\alpha {\lambda}_1},\sqrt{\alpha {\lambda}_2}$ and $\sqrt{\alpha {\lambda}_3}$ appearing in equation (5) were calculated from the equation 2*D* = *aλ* derived in [80]. The value range for the cell diffusion coefficient *D* was chosen according to the estimations made in [99]. The quantity $\sqrt{\alpha {\lambda}_1}$ was set much higher than $\sqrt{\alpha {\lambda}_2}$ and $\sqrt{\alpha {\lambda}_3}$, such that the diffusion of live cancer cells occurs primarily along the first vector. Table 1 summarizes parameter values used for the simulations of this section. An initial population of 5 ∙ 10^5^ live cancer cells was placed in the center of the simulated region.

**Table 1:** Simulation parameters

Parameter	Symbol	Value	References and remarks
Lattice size	*N*	21	
Time step	*Δτ*	10 s	
Voxel edge	*Δs*	2 mm	
Average cell population capacity for each voxel	*M*	8 ∙ 10^6^ cells	[74]
Maximum cell population capacity for each voxel	*M*_*max*_	1.02*M*	Assumed (see also appendix)
Oxygen Diffusion Coefficient	*D*_*o*_	1.8 ∙ 10^−5^ cm^2^/sec	[14, 52, 53]
Glucose Diffusion Coefficient	*D*_*gl*_	1.05 ∙ 10^− 5^ cm^2^/sec	[14, 52, 53]
Cell diffusion Coefficient	*D*_*c*_	1.5 ∙ 10^− 8^ to 1.5 ∙ 10^− 6^ cm^2^/sec	[56, 99]
Maximum mitosis rate	*a*_*max*_	16 ∙ 10^− 6^ mitoses/cell/10 s	Corresponding to a doubling time of 5 days in ideal chemical conditions
Cell cycle duration	*cc*	24 h	[36, 37]
Quiescent host cell Oxygen consumption	*K*_*o*_	250 ∙ 10^− 6^ pmol/sec	[36, 37, 84]
Quiescent host cell Glucose consumption	*K*_*gl*_	50 ∙ 10^− 6^ pmol/sec	[36, 37, 84]
Maximum absolute value of oxygen provision/absorption by local vasculature in normal tissue	*o*_*b*_*max*	2.8 ∙ 10^3^ pmol/sec	Estimated by values given in [93]
Maximum absolute value of glucose provision/absorption by local vasculature in normal tissue	*gl*_*b*_*max*	560 pmol/sec	Estimated by values given in [93]
Maximum vasculature expansion rate	*v*_*e*_	1.6 ∙ 10^−5^-8 ∙ 10^− 5^	Corresponding to minimum doubling time ranging from 5 to 1 days
Maximum vasculature regression rate	*v*_*r*_	1.6 ∙ 10^−5^-8 ∙ 10^− 5^	Corresponding to minimum halftime ranging from 5 to 1 days
Hypoxia threshold percentage	*h*_*o*_	30% of oxygen level in normal tissue	[5, 37]
Hypoglycemia threshold percentage	*h*_*gl*_	50% of glucose level in normal tissue	[37, 98]
Oxygen per voxel in normal tissue	$\overline{o_0}$	1.2 ∙ 10^3^ pmol	[45, 92] corresponding to concentration in capillary blood.
Glucose per voxel in normal tissue	$\overline{gl_0}$	40 ∙ 10^3^ pmol	[45, 92] corresponding to concentration in capillary blood.

Figs. 3 and 4 depict the tumor at the 70th and 90th day of the simulation, respectively. A general observation, holding for all performed simulations was that the chosen cell diffusion tensor affects the overall tumor shape in a noticeable way. This is depicted in Fig. 3, where the tumor shape is roughly similar to the ellipsoid of the diffusion tensor defined above. It can also be seen in Fig. 4, where the depicted tumor appears to be an approximately conformal expansion of the tumor in Fig. 3, although markedly distorted by the underlying stochasticity.

In Figs. 5 and 6, vertical sections of these tumors are shown, taken at the central (11th) voxel plane of the lattice. All colored voxels have been reached by the tumor, i.e. they contain a nonzero population of live plus necrotic cancer cells. We do not show voxels containing only host necrotic cells, something that was often observed in the near vicinity of the tumor periphery. We remind the reader that *M* is the average cell population capacity per voxel. The color code is as follows. Cyan voxels contain a total population of live tumor, necrotic tumor and necrotic host cells that does not exceed 50% of *M*. Where this quantity is above 50% of *M*, voxels are colored blue, gray or black, depending on the amount of necrotic cells they contain. Specifically, in blue voxels, the total necrotic (tumor+host) cell population is below 65% of *M*. In gray voxels, the total necrotic cell population is between 65 and 95% of *M*. In black voxels, necrotic cells are above 95% of *M*. Note that in both Figs. 5 and 6 necrosis is higher towards the tumor center. This is in agreement with the general observation that after some period of growth, due to vasculature disorganization and limited diffusion of nutrients near the tumor center, a necrotic core is formed; viable proliferating cells are located mainly at the outer rims of a tumor.

Figs. 7 and 8 depict a profile of the oxygen levels per voxel, corresponding to the vertical sections shown in Figs. 5 and 6, respectively. The darker the voxel shade, the lower the oxygen quantity in the specific voxel. The lightest shade indicates a voxel whose oxygen level is at least equal to normal. The darkest shade indicates a voxel whose oxygen level is below 15% of normal tissue level. The same color code holds for Figs. 9 and 10, which depict the respective glucose levels. Note that the tumor interior contains both hypoxic and hypoglycemic regions. From the simulations performed, hypoxic regions appeared to be much more spatially inhomogeneous and time varying than hypoglycemic ones, which generally tended to appear more congruent with necrotic areas of the tumor and overall tumor shape. Other than that, the shape of both hypoxic and hypoglycemic regions appeared to be largely random, and no pertinent spatial patterns were detected.

Similarly, Figs. 11 and 12 depict a similar profile of *o*_*b*_*max*_*t*_ for each voxel, corresponding respectively to the vertical sections shown in Figures 5 and 6. We remind the reader that, for a voxel *A*, *o*_*b*_*max*_*t*_(*A*) quantifies the capacity of the vascular network within *A* to provide/absorb oxygen to/from the surrounding tissue, thereby reflecting the regressed or expanded vasculature in *A* (see also section VI). The color code for Figs. 11 and 12 is as follows: The darkest grayscale shade indicates that for the respective voxel *A*, the value of *o*_*b*_*max*_*t*_(*A*) is below 50% of its value in normal tissue. The lightest grayscale shade indicates that for the respective voxel *A*, the value of *o*_*b*_*max*_*t*_(*A*) is equal to its value in normal tissue. Magenta indicates that *o*_*b*_*max*_*t*_(*A*) is between 100 and 125% of its value in normal tissue. Blue indicates that *o*_*b* _*max*_*t*_(*A*) is between 125 and 150% of its value in normal tissue. We note that since *o*_*b*_*max*_*t*_ and *gl*_*b*_*max*_*t*_ evolve temporally in the same way (see section VI), using the same color code yields identical figures for *gl*_*b*_*max*_*t*_. Figures 11 and 12 depict that towards the tumor center, *o*_*b*_*max*_*t*_ is lower, i.e. vasculature appears to be more regressed. Tumor periphery displays values larger than normal, reflecting the fact that at the outer rims of the tumor, angiogenesis takes place at a much faster rate than vessel regression.

As a more general use case, we performed a multivariate analysis on the effects of certain tumor growth related parameters on the number of viable tumor cells after a period of free growth. Specifically:

- Maximum mitosis rate for cancer cells *a*_*max*_ was fixed such that the corresponding minimum doubling time is 5 days.
- Parameter *λ* was varied in the set {2,4,6,8,10}. We remind the reader that *λK*_*ATP*_ is the ATP consumption rate of an actively proliferating tumor cell, where *K*_*ATP*_ is the ATP consumption rate of a normal host cell.
- The parameters *β*_1_ and *β*_2_, i.e. the minimum and maximum values of the energy fraction cancer cells acquire by glycolysis were varied in the set [*β*_1_ *β*_2_] ={[0 0.1], [0.1 0.2], [0.2 0.3], [0.3 0.4], [0.4 0.5]}
- Vasculature regression minimum halftime (in days) was varied in {1,2,3,4,5}.
- Vasculature expansion minimum doubling time (in days) was varied in {1,2,3,4,5}. The case where no angiogenesis occurred throughout the entire simulation was also considered.

**Fig. 13:**
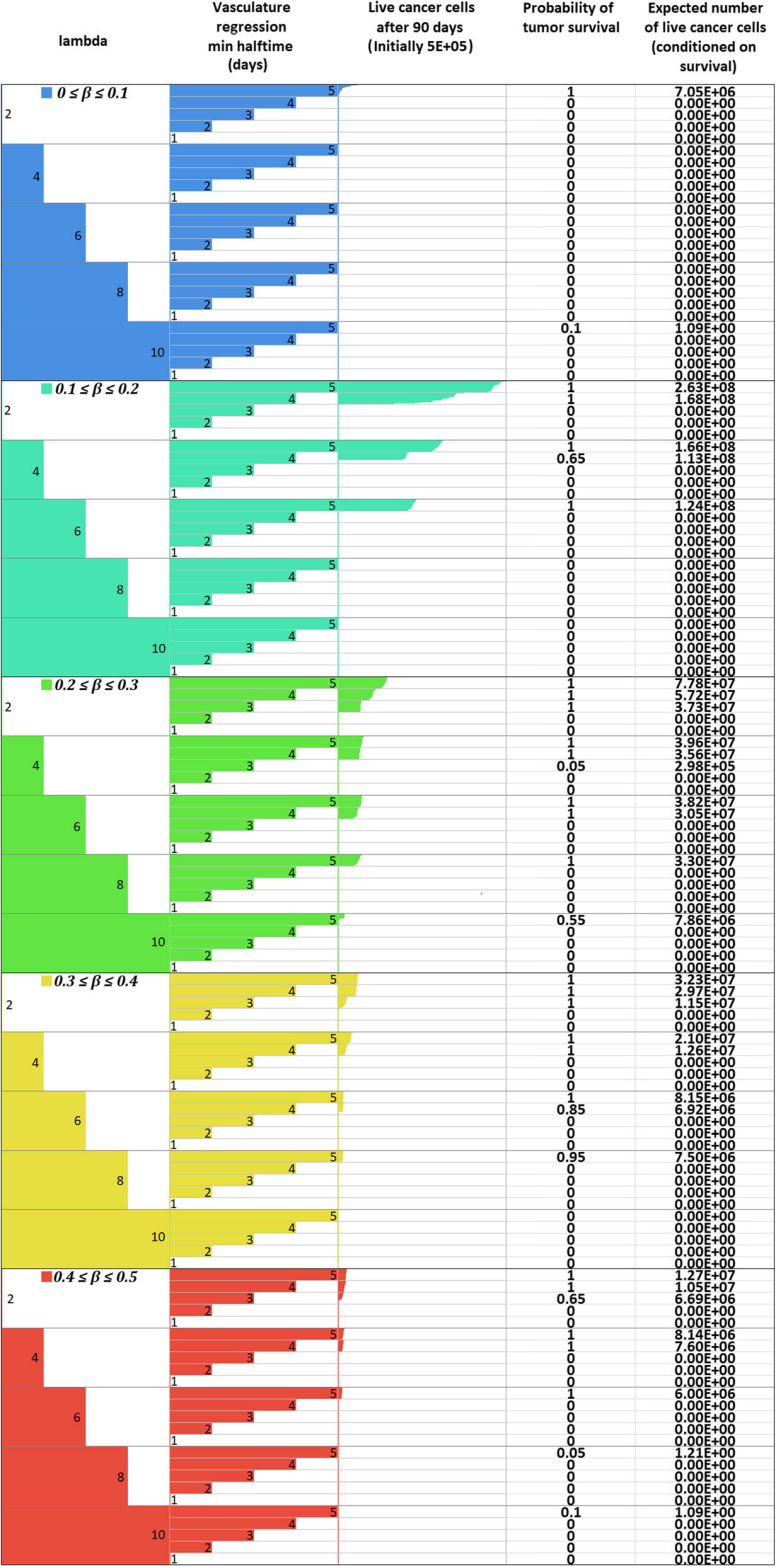
Tumor growth absent angiogenensis

**Fig. 14:**
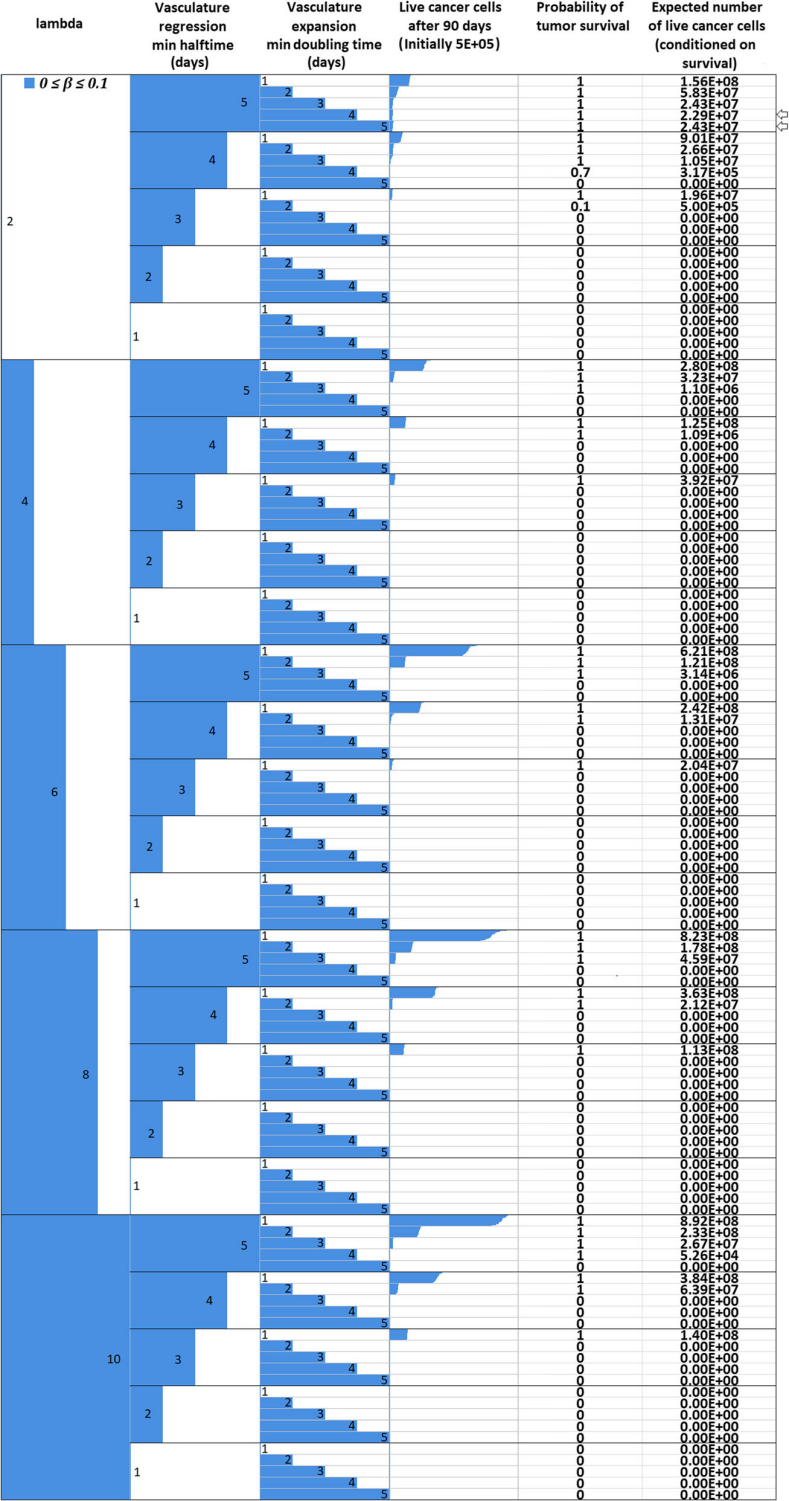
Tumor growth for 0 ≤ *β* ≤ 0.1, i.e. the tumor acquires energy mainly through combustion of glucose

**Fig. 15:**
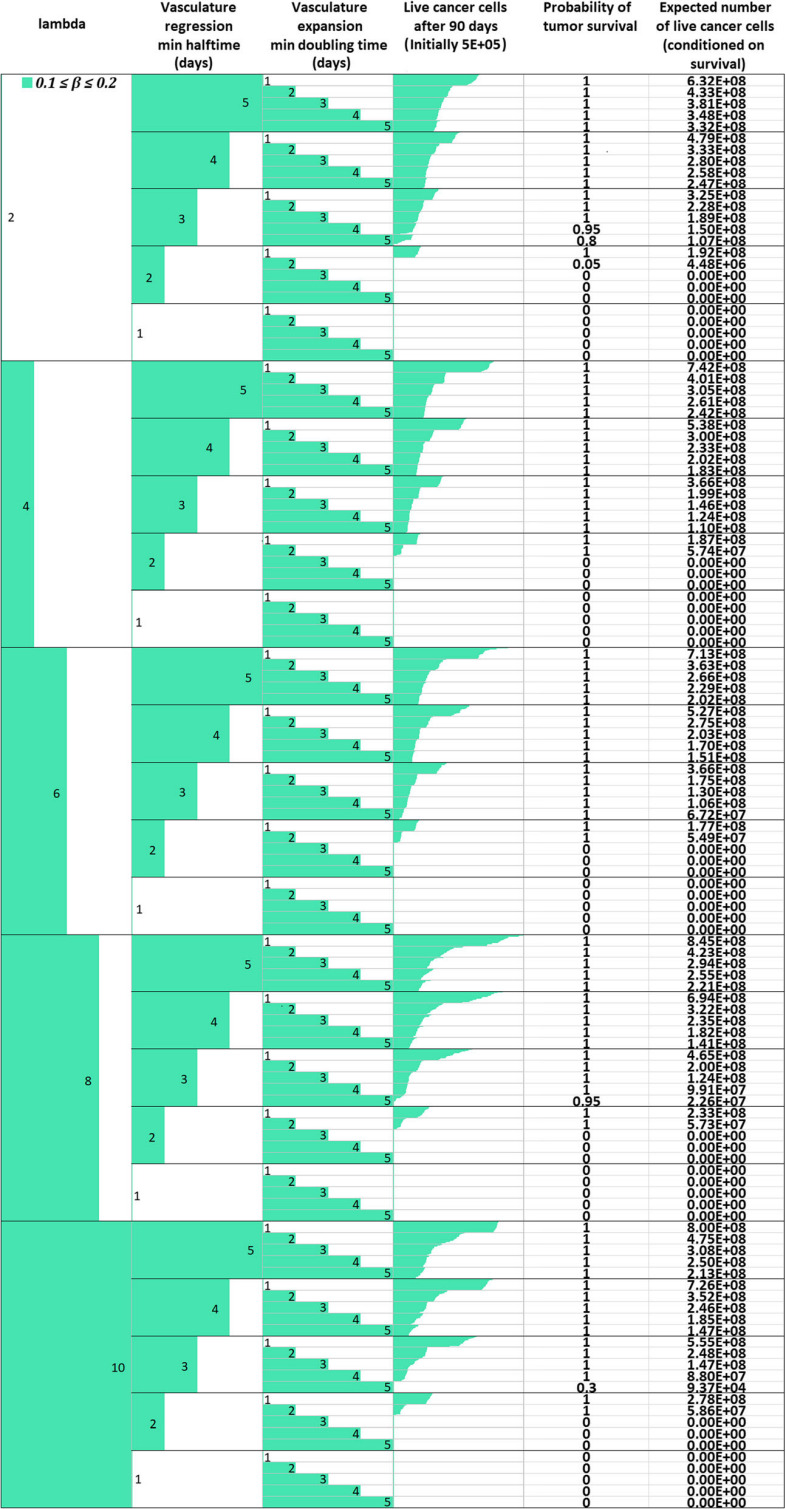
Tumor growth for 0.1 ≤ *β* ≤ 0.2, i.e. glycolysis is utilized more that in the respective cases of Figure 14

**Fig. 16:**
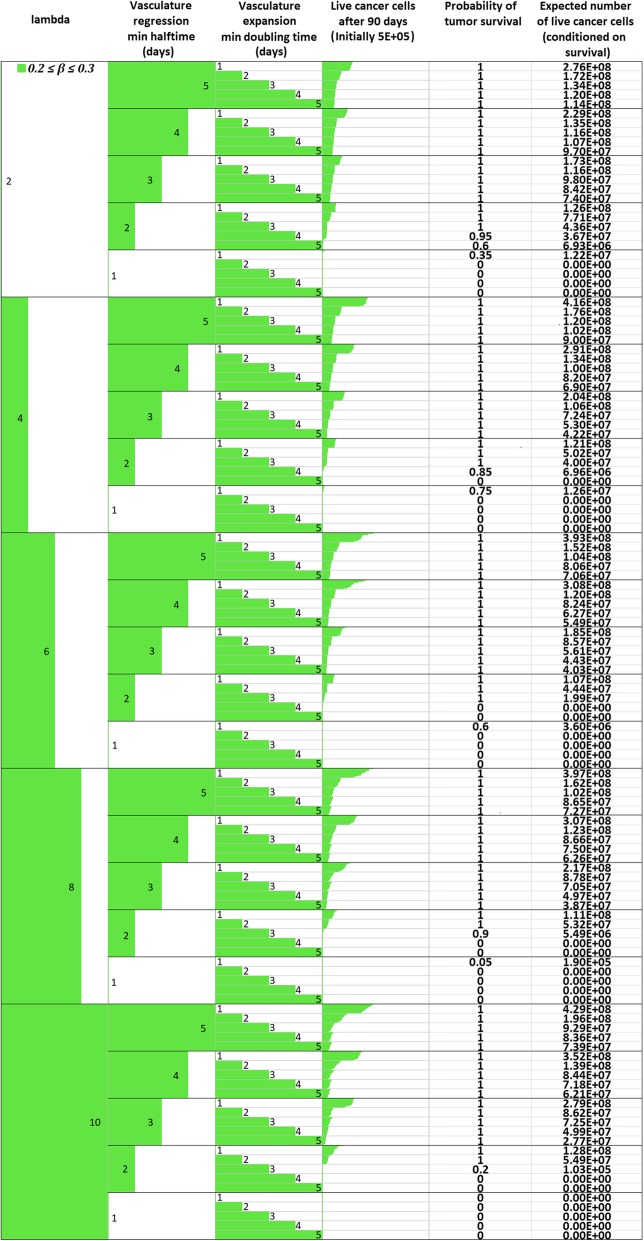
Tumor growth for 0.2 ≤ *β* ≤ 0.3, i.e. glycolysis is utilized more that in the respective cases of Fig. 15

**Fig. 17:**
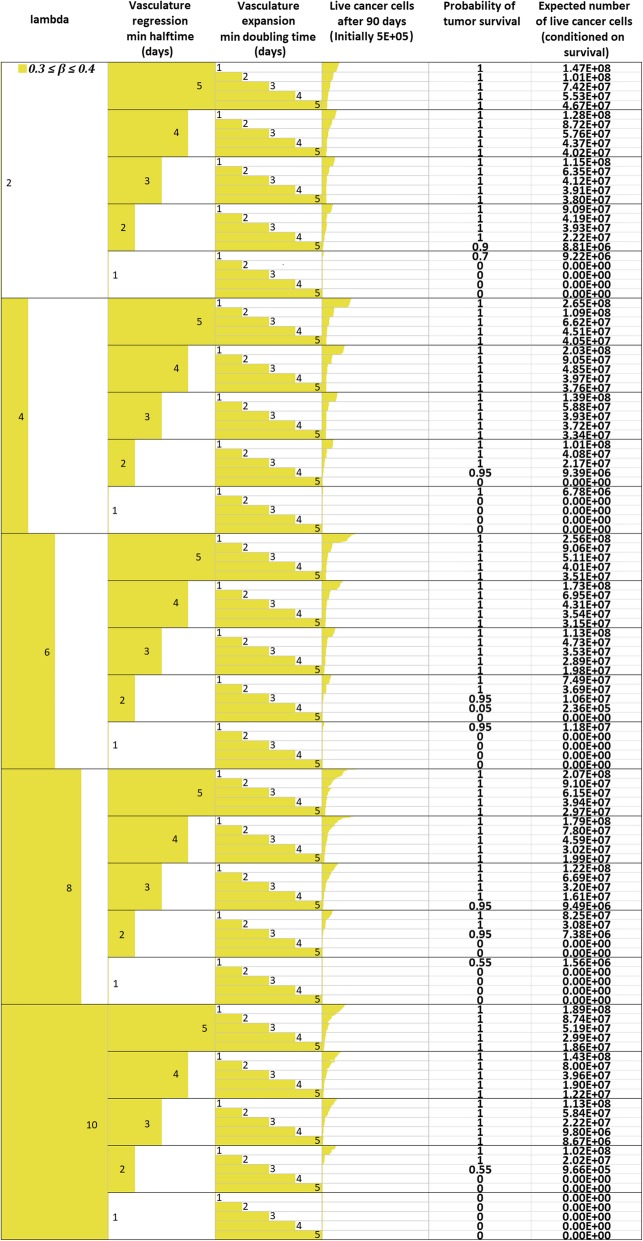
Tumor growth for 0.3 ≤ *β* ≤ 0.4, i.e. glycolysis is utilized more that in the respective cases of Fig. 16

**Fig. 18:**
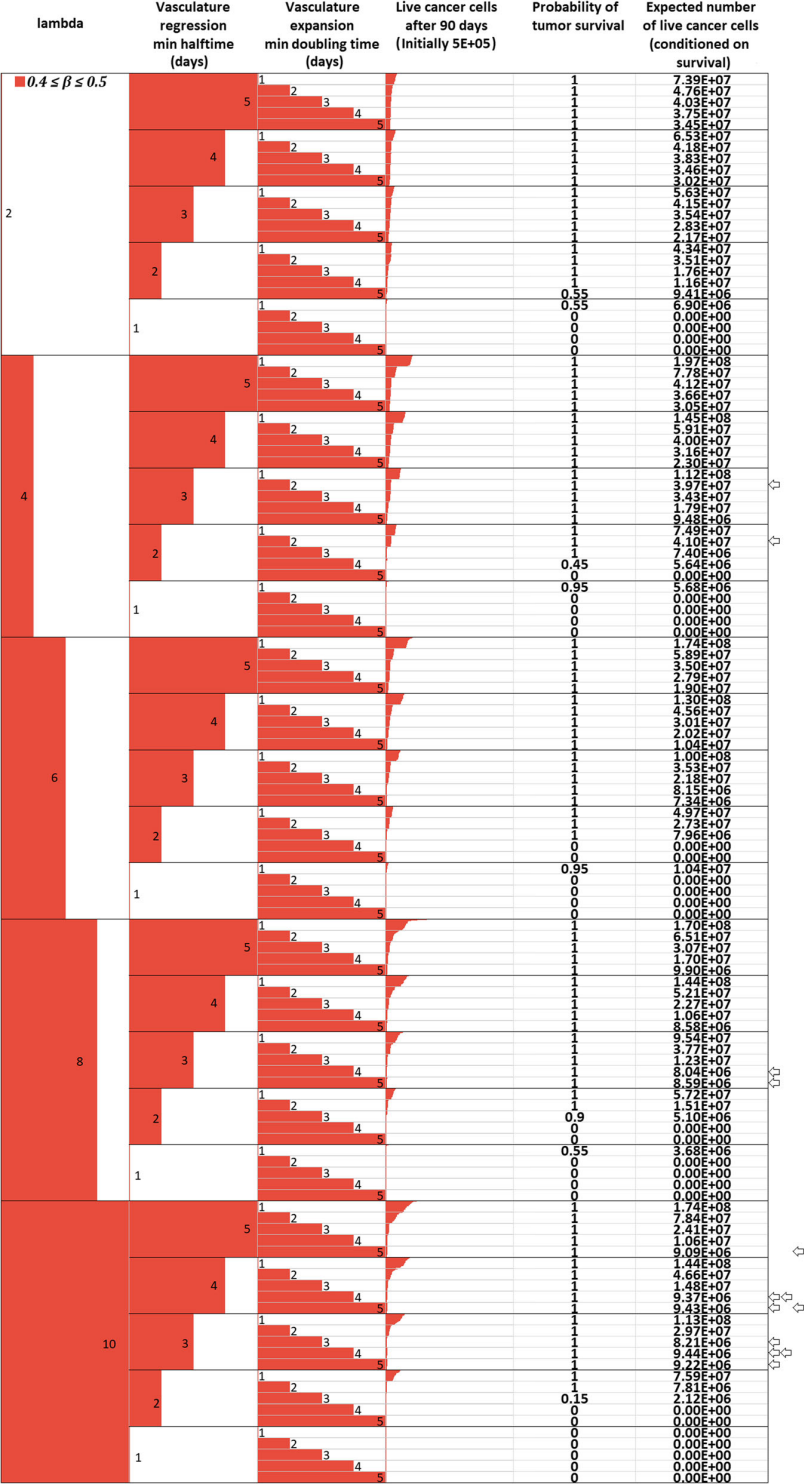
Tumor growth for 0.4 ≤ *β* ≤ 0.5, i.e. glycolysis is utilized more that in the respective cases of Fig. 17

The initial tumor population was 5 ∙ 10^5^ live cancer cells. The evolution of these tumors was simulated for a time period of 90 days. Due to the stochasticity of the model, for each set of parameter values a total of 20 simulations was performed. The results of these simulations are shown in Figs. 13, 14, 15, 16, 17, and 18.

In each of the Figs. 13, 14, 15, 16, 17, and 18, the columns ‘lambda’,‘Vasculature regression minimum halftime’ and ‘Vasculature expansion minimum doubling time’ are self-explanatory. Each row of the column ‘Live cancer cells after 90 days’ depicts the respective numbers of live tumor cells for each one of the 20 simulations performed for the parameters specified in the previous columns of the same row. In each row, these 20 numbers are drawn as horizontal lines, each one with length proportional to the resulting number of live cancer cells after 90 days. These 20 lines are drawn in sorted order with regard to their length, from longest (top) to shortest (bottom), and form the skewed bar observed in each row of the ‘Live cancer cells after 90 days’ column. We note that due to the smaller final populations observed absent angiogenesis, the aforementioned lines in Fig. 13 are drawn in a different scale than in Figs. 14, 15, 16, 17, and 18. In each row of the column ‘Probability of tumor survival’, we provide the fraction of simulations (out of the 20 simulations performed for the parameters of the specific row) that resulted to a nonzero population of live cancer cells. Each row of the column ‘Expected number of live cancer cells’ provides the expected number of live cancer cells after 90 days, conditioned on the survival of the tumor. This is essentially the mean of the resulting final populations, calculated by taking into account only the simulations that resulted in a nonzero final population of live cancer cells.

As explained above, each row of the ‘Live cancer cells after 90 days’ column depicts a skewed bar, formed by the final populations of live cancer cells for each of the 20 simulations performed for the specific row. Thus, the skewness of each such bar indicates the variance observed in the results of the respective simulations. For each such bar, a flatter right end indicates a lower variance; a more skewed right end indicates a higher variance. Visual inspection of these bars indicates that in most cases (i.e. rows), 20 simulations can provide a reasonable overview of the potential outcomes. However, there are cases where the skewness of the aforementioned bars is quite high, indicating a higher variance in the potential outcomes of the respective simulations. In such cases, like for example, the case in Fig. 13 where 0.1 ≤ β ≤ 0.2, lambda = 2 and Vasculature regression minimum halftime = 4 days or the same case but with lambda = 4, it is evident more simulations are needed. In general, our analysis showed that simulation parameters have an effect not only on the probability of survival and expected populations of cancer cells, but also, in several cases, on the variance of these populations. This was also observed for intermediate time points, i.e. cell populations calculated at time points within the overall time frame of 90 days.

Fig. 13 depicts the results of these simulations when tumors grow without angiogenesis. We see that the limits of the energy fraction *β* cancer cells can acquire from glycolysis play a crucial role on tumor growth and survival. For 0 ≤ *β* ≤ 0.1, i.e. when cells employ mainly combustion of glucose, tumors survive essentially only if they have minimal energy needs compared to host cells, and additionally, if vasculature regression evolves at a minimal rate. For 0.1 ≤ *β* ≤ 0.2, tumors survive in more cases than for 0 ≤ *β* ≤ 0.1 and in these cases they reach large end populations of viable cancer cells. For 0.2 ≤ *β* ≤ 0.3 tumors survive in even more cases, but don’t reach end populations as large as for 0.1 ≤ *β* ≤ 0.2. For 0.3 ≤ *β* ≤ 0.4 tumors survive almost like in the case where 0.2 ≤ *β* ≤ 0.3, although with lower probabilities and lower end populations. The same trend is observed when moving to the last case; for 0.4 ≤ *β* ≤ 0.5 tumors survive in slightly less cases than for 0.3 ≤ *β* ≤ 0.4, with lower probabilities and lower end populations. In each separate case, maximum vasculature regression rate (i.e. minimum halftime) and cancer cell energy requirements affect both probability of survival and final number of viable cells. In fact, in each case, the higher the maximum rate of vasculature regression, the lower are both the survival probability and the viable end population. Furthermore, in each case, for the same maximum rate of vasculature regression, higher energy requirements by tumor cells imply lower survival probabilities and viable end populations.

Figures 14, 15, 16, 17, and 18 consider additionally the effects of vasculature expansion. Visual inspection of these Figures shows that the limits of *β* affect the results in a way reminiscent of the one observed for the no angiogenesis case in Fig. 13. In Fig. 14, where 0 ≤ *β* ≤ 0.1, i.e. energy is acquired mainly through combustion, tumors grow only if vasculature regression evolves sufficiently slower than angiogenesis. In Fig. 15 (0.1 ≤ *β* ≤ 0.2), tumors survive and grow in many more cases, and in general they reach larger end populations of viable cells. In Fig. 16 (0.2 ≤ *β* ≤ 0.3), tumors survive in even more cases, however, end populations are lower than in Fig. 15. In terms of survival probabilities, tumors in Fig. 17 (0.3 ≤ *β* ≤ 0.4) tumors are slightly better than in Fig. 16, achieve, nevertheless, lower end populations. In Fig. 18 (0.4 ≤ *β* ≤ 0.5) tumors survive almost like in Fig. 17. Again, compared to Fig. 17, survival probabilities and end populations are lower.

A general observation is that, for the same limits of *β* and the same energy requirements *λ*, maximum rates of vasculature expansion and regression had monotonic effects on survival probabilities and viable end populations. Assuming other parameters equal, a higher maximum rate of vasculature expansion generally implies higher survival probability and viable end population. Respectively, a higher maximum rate of vasculature regression generally has the opposite effects. There are a few exceptions in these rules; they are marked by arrows at the left of each image and we will explain them below.

**Fig. 19:**
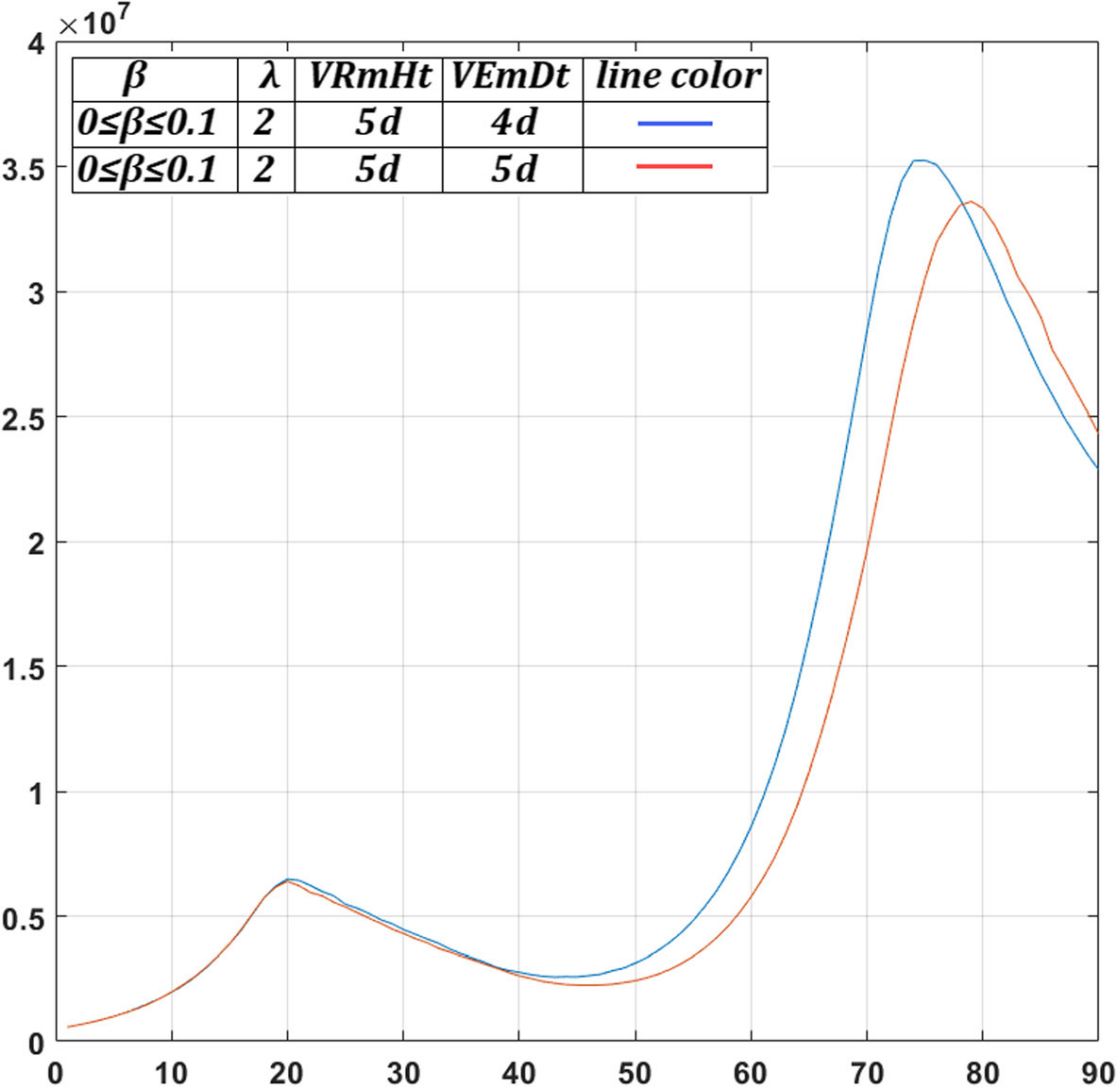
Timely evolution of live cancer cells for different vasculature regression and expansion rates. VRmHt: Vasculature Regression minimum Halftime, VEmDt: Vasculature Expansion minimum Doubling time, d: days

**Fig. 20:**
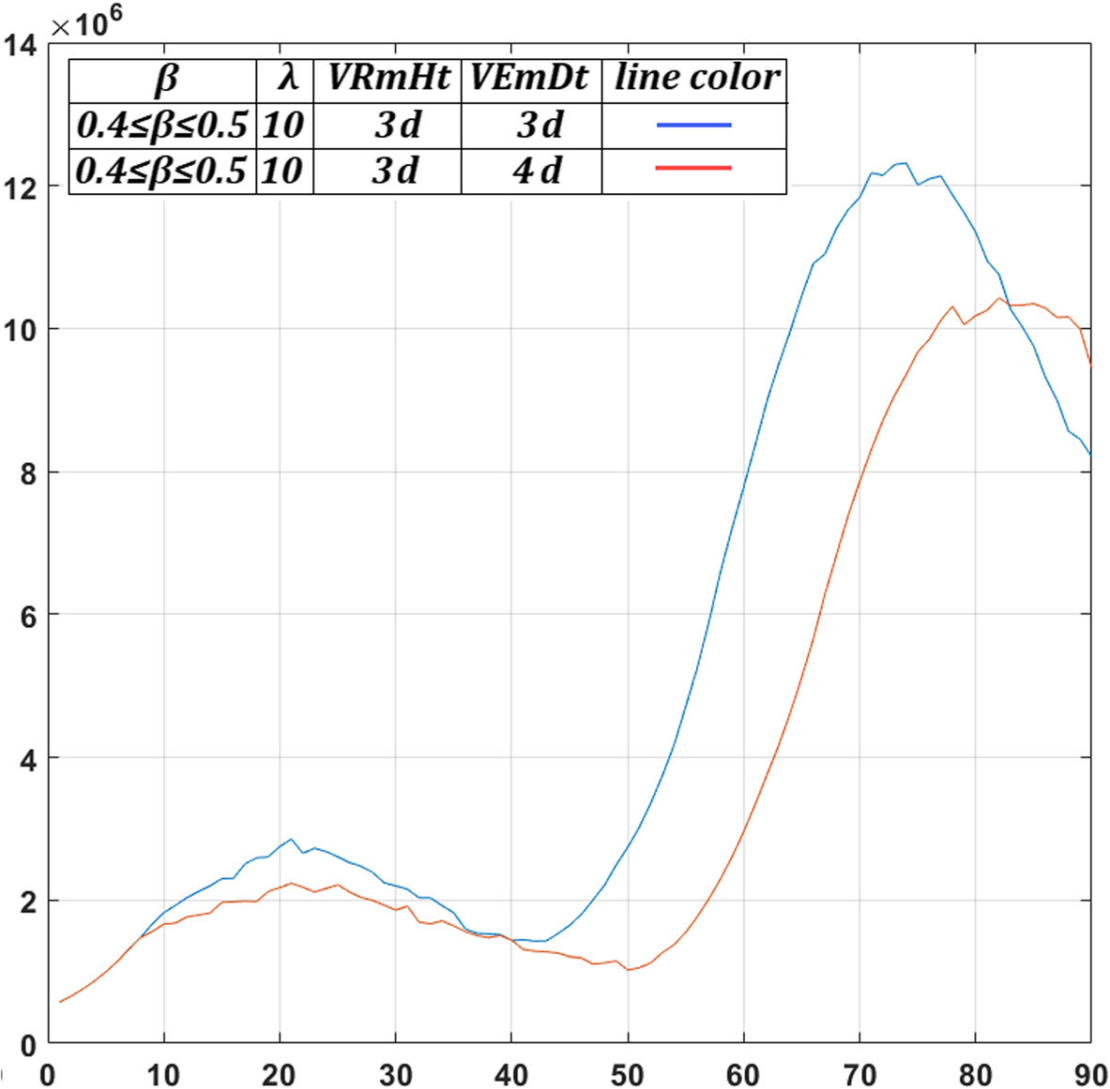
Timely evolution of live cancer cells for different vasculature regression and expansion rates. VRmHt: Vasculature Regression minimum Halftime, VEmDt: Vasculature Expansion minimum Doubling time, d: days

**Fig. 21:**
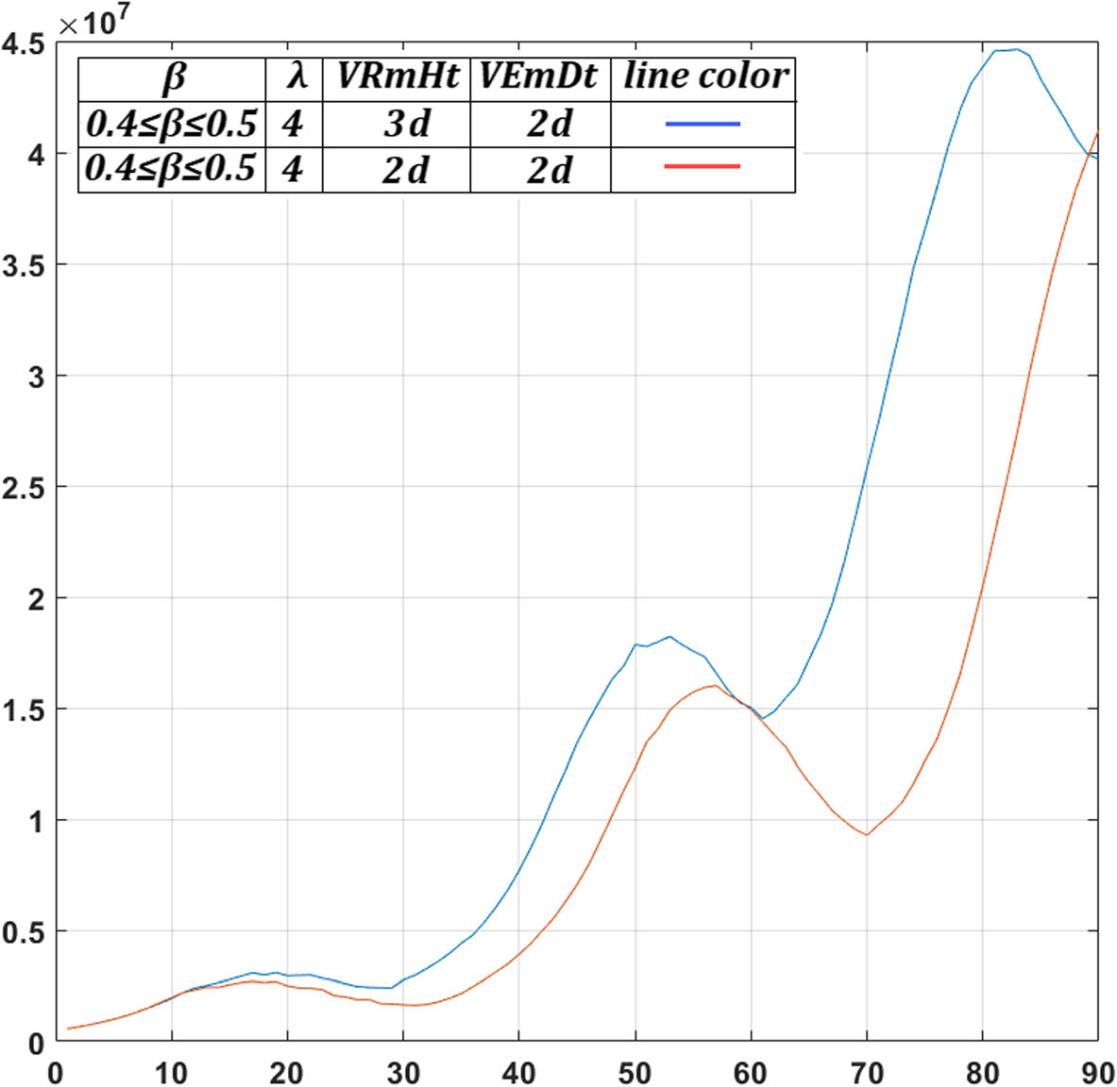
Timely evolution of live cancer cells for different vasculature regression and expansion rates. VRmHt: Vasculature Regression minimum Halftime, VEmDt: Vasculature Expansion minimum Doubling time, d: days

For all of the aforementioned exceptions, the expected number of live cancer cells was calculated for each day. These quantities were plotted in pairs for each case monotonicity did not hold; three such examples are shown in Figs. 19, 20, and 21. These Figures depict the pattern observed for all these cases. The expected behavior of tumor cells growing in more favorable conditions (blue curves) is to grow faster and larger than their respective counterparts (red curves) for most of the observed time period. However, they reach a maximum and start to regress sooner than tumor cells growing in less favorable conditions. Apparently though, on the 90th day, the sum of viable and necrotic tumor cells is larger for tumors growing in more favorable conditions.

The effects of *λ* (i.e. the parameter quantifying the energy requirements of cancer cells compared to host cells) are much more complicated. Using the abbreviations Vasculature Regression minimum Halftime (VRmHt) and Vasculature Expansion minimum Doubling time (VEmDt) defined for Figs. 19, 20 and 21, we observe the following. For 0 ≤ *β* ≤ 0.1, VRmHt = 5 days, VEmDt = 1 day, *λ* has an increasing effect on the viable end population. Apparently, in this case, high energy requirements induce hypoxia and hypoglycemia much more frequently, thereby triggering vessel expansion more often. Additionally, tumor-induced angiogenesis is much faster than tumor-induced vasculature regression; this results in higher final populations of viable cancer cells. The observation that, if angiogenesis occurs sufficiently faster than vascular regression, higher energy requirements act increasingly on the viable end population holds in general for 0 ≤ *β* ≤ 0.3, see Figs. 14, 15, and 16 . It is much less pronounced for 0.4 ≤ *β* ≤ 0.5 (Fig. 18), and does not hold for 0.3 ≤ *β* ≤ 0.4 (Fig. 17). As a counterexample, note that for 0.2 ≤ *β* ≤ 0.3, VRmHt = 5 days, VEmDt = 3 days, *λ* has a decreasing effect on the viable end population.

From the analysis performed so far, no general pattern providing a quantitative and concise interpretation of these observations was found. Evidently, the dependencies of the end population of viable cells on the parameters under consideration are quite complex, and display a rich structure of local maxima and minima. A more detailed study of these dependencies would require more simulations for each set of parameter values and additional consideration of standard deviations; we leave this for future work.

## Data Availability

Data sharing is not applicable to this article as no datasets were generated or analysed during the current study. All parameter values required to reproduce the presented simulations are included in this published article.

## Appendix

IX.i The random perturbation of matrices *T*_*o*_ and *T*_*gl*_ described in part III of the methods section is implemented as follows. There are two parameters involved

- *κ*, that is, the number of time steps every which the perturbation is applied to the matrices *T*_*o*_ and *T*_*gl*_ calculated by algorithm 2.
- *μ*, which is a number ranging from 0 to 9.

Every *κ* time steps, the respective columns of matrices *T*_*o*_ and *T*_*gl*_ are perturbed in the following way. Each nonzero probability of the column to be perturbed is multiplied by a uniformly distributed random number ranging from 1 to 1 + *μ*. There are 27 nonzero probabilities in each column, hence, we use 27 such random numbers. We then normalize these 27 products to sum to 1. Note that every time we apply this process on a column, the resulting ratio of any two of the column’s 27 probabilities could be anywhere between 1/(1 + *μ*) smaller and 1 + *μ* larger than its original value. Thus, their relative order of magnitude is preserved.

Sample simulations were performed with time step *Δτ *= 10 s, *κ *= 30, 60 (i.e. perturbations applied every 5 or 10 min) and *μ* = 1, 4, 9. The resulting cell populations did not vary significantly. In fact, they displayed an almost exact agreement on the numbers corresponding to their greatest order of magnitude and slight variations on the number corresponding to their second greatest order of magnitude. For the simulations described in section VIII, the values *κ *= 30 and *μ* = 1 were used.

IX.ii In this last part, we provide some suggestions on how the cellular diffusion module (section IV) may be re-adjusted, in order to consider slightly different sets of starting assumptions. We do this by discussing four specific examples, and show that each of them can be implemented using the sparse matrix, vector algebraic framework we propose. The resulting algorithmic modules were not used for a comprehensive analysis like the one presented in the main text. In this paper, they serve mainly as ideas on how to further elaborate on the specific module.

- For the simulations in the main text *M*_*max*_ was treated as a constant, and was set 2% larger than *M* for all voxels. A more general assumption would be to assume that for each voxel *A* the corresponding number *M*_*max*_(*A*) is a random number ranging close to *M*, that also changes with time; The corresponding implementation is straightforward: each time before applying Algorithm 2, generate this vector of random numbers; Then simply apply Algorithm 2, by first substituting $\overline{M_{max}}$ with this vector.
- Additionally to the assumptions (i)-(v) in section IV consider the following:
  (vi) Live cancer cells do not invade voxels whose total necrotic (cancer+normal) cell population is above *M*.
  This is an additional scenario that can be quite easily implemented in the sparse matrix framework we propose; each time before applying Algorithm 2 find all (new) voxels whose total necrotic population is above *M*. For each one of these voxels, do the following:

- Find the row of the matrix *T*_*c*_ corresponding to the voxel.
- Find the row’s nonzero elements;
- Set all row elements to zero;
- Normalize the columns of *T*_*c*_ corresponding to the nonzero elements of the row;

Subsequently, apply Algorithm 2 by using the resulting form of the matrix *T*_*c*_

c) Alternatively, instead of the previous additional assumption, consider the following:

(vi) Live cancer cells invading voxels whose total necrotic (cancer+normal) cell population is above *M* turn immediately to necrotic.

The implementation of the corresponding algorithm is a slight variation of Algorithm 2. We remind that for a vector *x* and a number *μ*, the vector (*x* ≥ *μ*) is the binary vector with elements set to 1 if the corresponding element of *x* is ≥*μ* and 0 otherwise. For vectors *x*, *y*, *x*. ∗ *y* denotes their elementwise product. The resulting algorithm is:

$$ {s}_0=\overline{M_{max}}-{nc}_t-{nn}_t $$

$$ {s}_1=\left({s}_0\ge 0\right).\ast {s}_0 $$

$$ {s}_2=\left(\left({w}_t>\overline{M_{max}}\ \right).\ast {s}_1\right)+\left(\left({w}_t\le \overline{M_{max}}\ \right).\ast {l}_t\right) $$

$$ {l}_{t+\varDelta \tau}={s}_2+\left(\left({nc}_t+{nn}_t\right)<M\right).\ast \left({T}_c\ast \left({l}_t-{s}_2\right)\right) $$

$$ {nc}_{t+\varDelta \tau}={nc}_t+\left(\left({nc}_t+{nn}_t\right)\ge M\right).\ast \left({T}_c\ast \left({l}_t-{s}_2\right)\right) $$

Note that in the last two lines, invading cells are added to the live cancer cells of the invaded voxel if its total necrotic population is below *M*. If its total necrotic population is above *M*, invading cells are added to the necrotic cancer cells of the voxel.

d) Last, we informally discuss some ideas on how to introduce some at least crude, phenomenological notion of chemotaxis in this module. Chemotaxis refers to the phenomenon where cells tend to move towards higher concentrations of specific molecules, say, for example, oxygen. We remind that each column of the matrix *T*_*c*_ corresponds to a voxel *A*. Each nonzero element of the column corresponds to a specific neighbor of *A*. In fact, each nonzero element is the fraction of live cancer cells in *A* that are in excess of *M*_*max*_ that will invade the respective neighbor. How can we quantify the tendency of cells to move towards higher concentrations of oxygen in this framework? The simplest, although admittedly crude way is the following. Each time before executing Algorithm 2, for each voxel that has been reached by the tumor or is adjacent to it, multiply elementwise the respective column with the current vector of oxygen quantities *o*_*t*_. Then normalize its resulting nonzero elements (which remain in the same place) to sum to one. This process rescales the probability of invading each neighbor according to its respective oxygen content; We stress that this is merely a rough idea, which however, we wanted to share. The quantitative details and overall soundness of this idea remain to be furtherly analyzed and improved.

## Abbreviations

DTI: Diffusion Tensor Imaging
PET: Positron Emission Tomography
VEmDt: Vasculature Expansion minimum Doubling time
VRmHt: Vasculature Regression minimum Halftime
