# Publisher Correction to: Three-dimensional tumor growth in time-varying chemical fields: a modeling framework and theoretical study

**DOI:** 10.1186/s12859-019-3102-0

**Published:** 2019-10-16

**Authors:** Markos Antonopoulos, Dimitra Dionysiou, Georgios Stamatakos, Nikolaos Uzunoglu

**Affiliations:** Institute of Communication and Computer Systems, National Technical University of Athens, Athens, Greece

**Correction to: BMC Bioinformatics**

**https://doi.org/10.1186/s12859-019-2997-9**

Following publication of the original article [1], the authors noticed that the following errors were introduced by pdf/html formatting issues. The original article has been corrected. The publisher apologizes to the authors and readers for these errors.

***Page 9, first column:***

The paragraph

“- *o b*_*t*_(*A*): sec) by the local vascular network within/sec) by the local vascular network within *A* during the previous time interval *t* − *Δτ* → *t*”

Should be replaced with

“- *o*_*b*_*t*_(*A*) : Oxygen supply rate (pmols/sec) by the local vascular network within *A during* the previous time interval *t* − *Δτ* → *t* ”

In the subsequent paragraph, i.e. the paragraph starting starting with the phrase “ - *gl*_*b*_*t*_(*A*): Glucose supply rate … ” ,

the phrase “ interval−*Δτ* → *t* ” should be replaced with “ interval *t* − *Δτ* → *t* ”

***Page 11, second column:***

The equation $\beta \ge 1-\frac{6{O}_{av}}{l_t(A){K}_{ATP}\varDelta \tau}\equiv \beta \_$ should be $\beta \ge 1-\frac{6{O}_{av}}{l_t(A){K}_{ATP}\varDelta \tau}\equiv \underset{\_}{\beta }$ .

In the subsequent sentence, i.e. “Since *a*_*o*_(*β*) is increasing, *β*_ is actually … ” the “*β*_ ” should be “ $\underset{\_}{\beta }$ ”.

In the subsequent paragraph (first bullet point), in the sentence “ If *β* _  > *β*_2_, we have that … ” the “ *β*_ ” should be “ $\underset{\_}{\beta }$ ”.

In the subsequent paragraph (second bullet point), i.e. “ If *β* _  ≤ *β*_2_, we have that for each *β* ∈ [*max*(*β*_, *β*_1_), *β*_2_] it holds that *a*_*o*_(*β*) ≥ 0. ” all occurences of “ *β*_ ” should be “ $\underset{\_}{\beta }$ ”.

In paragraph 11a, first line, “ *a*_*o*_(*β*) ” should be “ *a*_*gl*_(*β*) ”.

***Page 12, first column:***

In the sentence “ Case 2.2.1. If *β* > *β*_2_ or $\overline{\beta}<{\beta}_1$ or $\mathit{\min}\left(\overline{\beta},{\beta}_2\right)<\mathit{\max}\left(\beta \_,{\beta}_1\right)$, the analysis above implies that … ” the inequality *β* > *β*_2_ should be $\underset{\_}{\beta }>{\beta}_2$ and the inequality $\mathit{\min}\left(\overline{\beta},{\beta}_2\right)<\mathit{\max}\left(\beta \_,{\beta}_1\right)$ should be $\mathit{\min}\left(\overline{\beta},{\beta}_2\right)<\mathit{\max}\left(\underset{\_}{\beta },{\beta}_1\right)$.

In the sentence “ Case 2.2.2. If *β* ≤ *β*_2_, $\overline{\beta}\ge {\beta}_1$ and $\mathit{\min}\left(\overline{\beta},{\beta}_2\right)\ge \mathit{\max}\left(\beta \_,{\beta}_1\right)$… ” the inequality *β* ≤ *β*_2_ should be $\underset{\_}{\beta}\le {\beta}_2$ and the inequality $\mathit{\min}\left(\overline{\beta},{\beta}_2\right)\ge \mathit{\max}\left(\beta \_,{\beta}_1\right)$ should be $\mathit{\min}\left(\overline{\beta},{\beta}_2\right)\ge \mathit{\max}\left(\underset{\_}{\beta },{\beta}_1\right)$ .

In the same paragraph, the mathematical expression $\beta \in \left[\mathit{\max}\left(\beta \_,{\beta}_1\right),\mathit{\min}\left(\overline{\beta},{\beta}_2\right)\right]$ should be $\beta \in \left[\mathit{\max}\left(\underset{\_}{\beta },{\beta}_1\right),\mathit{\min}\left(\overline{\beta},{\beta}_2\right)\right]$

***Page 12, second column:***

In the sentence “ Again, we pick a random $\overset{\sim }{\beta }$ in $\left[\mathit{\max}\left(\beta \_,{\beta}_1\right),\mathit{\min}\left(\overline{\beta},{\beta}_2\right)\right]$. ” the mathematical expression $\left[\mathit{\max}\left(\beta \_,{\beta}_1\right),\mathit{\min}\left(\overline{\beta},{\beta}_2\right)\right]$ should be $\left[\mathit{\max}\left(\underset{\_}{\beta },{\beta}_1\right),\mathit{\min}\left(\overline{\beta},{\beta}_2\right)\right]$

***Page 14, second column:***

The equations

$$ ob\ {\mathit{\max}}_{t+\varDelta \tau}(A)=\left(1-{f}_r\left({l}_t(A),{nc}_t(A)\right){v}_r+{sw}_t(A)\ {f}_e\left({l}_t(A),{nc}_t(A),{nn}_t(A)\right){v}_e\right)\bullet ob\ {\mathit{\max}}_t(A) $$

$$ glb\ {\mathit{\max}}_{t+\varDelta \tau}(A)=\left(1-{f}_r\left({l}_t(A),{nc}_t(A)\right){v}_r+{sw}_t(A){f}_e\left({l}_t(A),{nc}_t(A),{nn}_t(A)\right){v}_e\right)\bullet glb\ {\mathit{\max}}_t(A) $$

should be

$$ o\_b\_{\mathit{\max}}_{t+\varDelta \tau}(A)=\left(1-{f}_r\left({l}_t(A),{nc}_t(A)\right){v}_r+{sw}_t(A)\ {f}_e\left({l}_t(A),{nc}_t(A),{nn}_t(A)\right){v}_e\right)\bullet o\_b\_{\mathit{\max}}_t(A) $$

$$ gl\_b\_{\mathit{\max}}_{t+\varDelta \tau}(A)=\left(1-{f}_r\left({l}_t(A),{nc}_t(A)\right){v}_r+{sw}_t(A){f}_e\left({l}_t(A),{nc}_t(A),{nn}_t(A)\right){v}_e\right)\bullet gl\_b\_{\mathit{\max}}_t(A). $$

The equations

$$ ob\ {\mathit{\max}}_{t+\varDelta \tau}(A)=\left(1-{f}_r\left({l}_t(A),{nc}_t(A)\right){r}_3{v}_r+{sw}_t(A)\kern0.5em {f}_e\left({l}_t(A),{nc}_t(A),{nn}_t(A)\right){r}_4{v}_e\right)\bullet ob\ {\mathit{\max}}_t(A) $$

$$ glb\ {\mathit{\max}}_{t+\varDelta \tau}(A)=\left(1-{f}_r\left({l}_t(A),{nc}_t(A)\right){r}_3{v}_r+{sw}_t(A){f}_e\left({l}_t(A),{nc}_t(A),{nn}_t(A)\right){r}_4{v}_e\right)\bullet glb\ {\mathit{\max}}_t(A) $$

should be

$$ o\_b\_{\mathit{\max}}_{t+\varDelta \tau}(A)=\left(1-{f}_r\left({l}_t(A),{nc}_t(A)\right){r}_3{v}_r+{sw}_t(A)\kern0.5em {f}_e\left({l}_t(A),{nc}_t(A),{nn}_t(A)\right){r}_4{v}_e\right)\bullet o\_b\_{\mathit{\max}}_t(A) $$

$$ gl\_b\_{\mathit{\max}}_{t+\varDelta \tau}(A)=\left(1-{f}_r\left({l}_t(A),{nc}_t(A)\right){r}_3{v}_r+{sw}_t(A){f}_e\left({l}_t(A),{nc}_t(A),{nn}_t(A)\right){r}_4{v}_e\right)\bullet gl\_b\_{\mathit{\max}}_t(A) $$

***Page 15, first column:***

The equations

$$ ob\ {\mathit{\max}}_{t+\varDelta \tau}(A)=\left(1-\frac{l_t(A)+{nc}_t(A)}{M}{r}_3{v}_r+{sw}_t(A)\kern0.5em \frac{M-{l}_t(A)-{nc}_t(A)-{nn}_t(A)}{M}{r}_4{v}_e\right)\bullet ob\ {\mathit{\max}}_t(A) $$

$$ glb\ {\mathit{\max}}_{t+\varDelta \tau}(A)=\left(1-\frac{l_t(A)+{nc}_t(A)}{M}{r}_3{v}_r+{sw}_t(A)\frac{M-{l}_t(A)-{nc}_t(A)-{nn}_t(A)}{M}{r}_4{v}_e\right)\bullet glb\ {\mathit{\max}}_t(A) $$

should be

$$ o\_b\_{\mathit{\max}}_{t+\varDelta \tau}(A)=\left(1-\frac{l_t(A)+{nc}_t(A)}{M}{r}_3{v}_r+{sw}_t(A)\kern0.5em \frac{M-{l}_t(A)-{nc}_t(A)-{nn}_t(A)}{M}{r}_4{v}_e\right)\bullet o\_b\_{\mathit{\max}}_t(A) $$

$$ gl\_b\_{\mathit{\max}}_{t+\varDelta \tau}(A)=\left(1-\frac{l_t(A)+{nc}_t(A)}{M}{r}_3{v}_r+{sw}_t(A)\frac{M-{l}_t(A)-{nc}_t(A)-{nn}_t(A)}{M}{r}_4{v}_e\right)\bullet gl\_b\_{\mathit{\max}}_t(A) $$

***Page 15, second column:***

The equations

$$ ob\ {\mathit{\max}}_{t+\varDelta \tau}(A)=\left(1-\frac{l_t(A)+{nc}_t(A)}{M}{r}_3{v}_r+{sw}_t(A)\kern0.5em \frac{M-{l}_t(A)-{nc}_t(A)-{nn}_t(A)}{M}{r}_4{v}_e\right)\bullet ob\ {\mathit{\max}}_t(A) $$

$$ glb\ {\mathit{\max}}_{t+\varDelta \tau}(A)=\left(1-\frac{l_t(A)+{nc}_t(A)}{M}{r}_3{v}_r+{sw}_t(A)\frac{M-{l}_t(A)-{nc}_t(A)-{nn}_t(A)}{M}{r}_4{v}_e\right)\bullet glb\ {\mathit{\max}}_t(A) $$

Should be

$$ o\_b\_{\mathit{\max}}_{t+\varDelta \tau}(A)=\left(1-\frac{l_t(A)+{nc}_t(A)}{M}{r}_3{v}_r+{sw}_t(A)\kern0.5em \frac{M-{l}_t(A)-{nc}_t(A)-{nn}_t(A)}{M}{r}_4{v}_e\right)\bullet o\_b\_{\mathit{\max}}_t(A) $$

$$ gl\_b\_{\mathit{\max}}_{t+\varDelta \tau}(A)=\left(1-\frac{l_t(A)+{nc}_t(A)}{M}{r}_3{v}_r+{sw}_t(A)\frac{M-{l}_t(A)-{nc}_t(A)-{nn}_t(A)}{M}{r}_4{v}_e\right)\bullet gl\_b\_{\mathit{\max}}_t(A) $$

The second equation appearing in this column, i.e.

$$ gl\_{b}_{t+\varDelta \tau}(A)={B}_{o\_b\_{\mathit{\max}}_{t+\varDelta \tau}(A)}\left(\kern0.5em gl\_{b}_t(A)+{r}_2\left(\left(\ \overline{gl_0}-{gl}_t(A)\ \right)/\varDelta \tau \right)\kern0.5em \right) $$

Should be

$$ gl\_{b}_{t+\varDelta \tau}(A)={B}_{gl\_b\_{\mathit{\max}}_{t+\varDelta \tau}(A)}\left(\kern0.5em gl\_{b}_t(A)+{r}_2\left(\left(\ \overline{gl_0}-{gl}_t(A)\ \right)/\varDelta \tau \right)\kern0.5em \right) $$
